# Development of liquid culture media mimicking the conditions of sinuses and lungs in cystic fibrosis and health

**DOI:** 10.12688/f1000research.125074.1

**Published:** 2022-09-07

**Authors:** Dilem Ruhluel, Siobhan O'Brien, Joanne L Fothergill, Daniel R Neill

**Affiliations:** 1 University of Liverpool, Institute of Infection, Veterinary and Ecological Sciences,, Liverpool, L69 7BE, UK; 2 Department of Microbiology, Moyne Institute of Preventive Medicine, Trinity College, Dublin, 2, Ireland

**Keywords:** Cystic Fibrosis (CF), sinuses, lungs, in vitro models, 3Rs

## Abstract

The respiratory tract is a compartmentalised and heterogenous environment. The nasopharynx and sinuses of the upper airways have distinct properties from the lungs and these differences may shape bacterial adaptation and evolution. Upper airway niches act as early colonisation sites for respiratory bacterial pathogens, including those, such as *Pseudomonas aeruginosa*, that can go on to establish chronic infection of the lungs in people with cystic fibrosis (CF). Despite the importance of upper airway environments in facilitating early adaptation to host environments, currently available *in vitro* models for study of respiratory infection in CF focus exclusively on the lungs. Furthermore, animal models, widely used to bridge the gap between *in vitro* systems and the clinical scenario, do not allow the upper and lower airways to be studied in isolation. We have developed a suite of culture media reproducing key features of the upper and lower airways, for the study of bacterial adaptation and evolution in different respiratory environments. For both upper and lower airway-mimicking media, we have developed formulations that reflect airway conditions in health and those that reflect the altered environment of the CF respiratory tract. Here, we describe the development and validation of these media and their use for study of genetic and phenotypic adaptations in *P. aeruginosa* during growth under upper or lower airway conditions in health and in CF.

Research highlights **Scientific benefit(s):**

Allows:

• Study of bacterial pathogenicity in infection relevant conditions of compartmentalised airway niches
• Study of bacterial host adaptation in cystic fibrosis (CF) and other respiratory diseases

**3Rs benefit(s):**

• Reduces the need for vertebrates in respiratory microbiology by providing a pre-screening platform for bacterial pathogenicity and host-pathogen interaction related research questions

**Practical benefit(s):**

• Inexpensive, simple to operate, no training required
• Chemically defined and easy to manipulate the content of the media to study different research questions, with several respiratory pathogens

**Current applications:**

• Suitable for studying phenotypes including bacterial growth, antimicrobial resistance, biofilm formation,
• Suitable for studying bacterial gene expression in health and CF under relevant host stresses

**Potential applications:**

Offers a platform for:

• Studying long term evolution of bacteria in health and infection relevant media
• Studying different pathogen combinations and how they affect virulence and resistance
• Combining with cell or tissue-based models to fully capture the chemical and cellular characteristics of different airway niches in the lab environment
• Assessing efficacy of drugs and therapeutics under relevant conditions

## Introduction

Cystic Fibrosis (CF) is an autosomal recessive genetic disorder caused by mutations in the cystic fibrosis transmembrane conductance regulator ( *CFTR*) gene, located on the long arm of chromosome 7. ^1^ CFTR protein functions as an apical ion channel. ^1^ CF has a range of severities, with airway disease being the main cause of morbidity and mortality. ^1^ Defects in CFTR affect its ion transporting functions, leading to accumulation of sticky mucus on epithelial surfaces in affected organs, including the lungs, sinuses, pancreas, gastrointestinal tract, sweat glands and reproductive tract. ^1^

The warm, humid and nutrient rich environment of CF airways supports growth of various bacteria. *Pseudomonas aeruginosa* is one of the most commonly isolated bacterial pathogens of the airways of people with CF (PwCF). ^2^ *P. aeruginosa* is a Gram-negative aerobic/facultative anaerobic bacterium found in high abundance in external environments. ^2^ *P. aeruginosa* colonization and infection of the airways is a significant contributor to morbidity and mortality in PwCF. ^3^ When *P. aeruginosa* enters into the airways from an environmental source, it not only must adapt to the changes in nutritional and physiochemical status associated with the airway environment, but also to stresses exerted from intense antimicrobial pressure and the actions of immune responses, competition from resident microbiota and viscous mucus-related osmotic stresses. ^2^ All of these factors influence the adaptation and evolution of *P. aeruginosa* within respiratory niches. ^2^

*P. aeruginosa* has a >6 Mb ^4^ plastic genome that facilitates rapid adaptation to environmental stimuli. ^5^ Environmental versatility is achieved through the action of regulatory systems, which collectively make up ~8% of the *P. aeruginosa* genome. ^6^ Transition from colonization to acute infection and subsequent biofilm-associated chronic infection takes place as a result of changes in these regulatory systems that are key for controlling the expression of virulence factors in response to host environmental cues. ^7^

The upper respiratory tract (and particularly the paranasal sinuses) has been suggested to be a protective niche for environmentally-acquired *P. aeruginosa* during early infection. Colonisation of this environment is thought to precede chronic lung infection. ^8^^,^^9^ *P. aeruginosa* has been identified in sinusitis samples and a high level of similarity has been observed between paranasal and lung isolates. ^10^ The presence of a low number of immune cells and reduced exposure to antibiotics in the upper airways, relative to lungs, provides a transitional niche within which the bacteria can adapt to host conditions before establishing chronic lung infection. Early infection of lungs with environmental *P. aeruginosa* may be cleared by a combination of host inflammatory responses and antibiotic treatment, but the persistence of the pathogen within the upper airways offers opportunity for later downward reseeding of a host-adapted bacterial population. Armbuster *et al.* documented the role of the sinuses in *P. aeruginosa* within-host adaptation in 106 adult CF patients. They found that the sinuses harboured isolates with pathoadaptive characteristics that were previously seen in isolates from sputum and that have been associated with persistence in the CF lung. ^11^ The continuous cycle of bacterial clearance from lungs and recolonization from sinuses may form an important part of the intermittent colonization stage that precedes chronic lung infection. ^12^

This proposed route to lung infection was recapitulated in a mouse natural inhalation infection model with *P. aeruginosa*, where persistence of bacteria in the upper airways was observed over the course of 28 days of infection. Initial colonisation of lungs was cleared within two weeks of infection *,* in the absence of antibiotic treatment, but *P. aeruginosa* was isolated from sinuses throughout infection and reappeared in the lungs of all mice at day 28 post-infection, suggesting that long-term upper airway persistence may allow bacterial migration and reseeding to the lower airways. ^8^ In later studies, this within-host adaptation was linked to loss-of-function mutations in the two component regulatory system gene *pmrB,* thought to have been acquired during upper airway infection. ^13^ Isolates carrying *pmrB* mutations, recovered from the murine airways, showed enhanced potential for establishment and maintenance of lung infection that was linked to increased resistance to lysozyme and increased attachment to host surfaces. Similar loss-of-function mutations were identified in *pmrB* in clinical isolates from CF. As the experiments undertaken in mice did not involve antibiotic treatment, the selective pressure for the observed changes was hypothesised to be the result of the action of host-derived antimicrobials. ^13^

Other pathoadaptive characteristics of strains from chronic infections have been identified and include overproduction of alginate, reduction in growth rate, loss of motility, decreased susceptibility to antimicrobials and diversification in colony morphology. ^14^^–^^16^ Changes in colony morphologies, towards a so-called ‘wrinkly’ phenotype, were indicated to be the result of redox-drive adaptations to maximize oxygen accessibility in biofilm by increasing the surface area exposed to air. ^17^ A slow growing phenotype has also been suggested to be a common characteristic of long term CF airway colonization and is thought to develop as a result of continuous activation of stress responses driven by exposure to cells and molecules of the immune system. ^18^

The respiratory tract is a heterogenous, compartmentalised environment, with the biological, chemical and physical properties of each niche shaping *P. aeruginosa* adaptive evolution in different ways. A range of *in vitro* and *in vivo* models are available for the study of respiratory infection in CF. The most basic of these involve *in vitro* culture in nutrient rich laboratory media that readily supports bacterial growth. These models are often a poor reflection of the airway environment, as they contain sugars and proteins at concentrations that are not physiologically relevant and they lack important host-derived factors that exert stress on pathogens or that are recognised by microbial environmental sensing systems. These limitations encouraged the development of chemically-defined liquid culture media that better reflect the conditions of the CF airways. Artificial sputum media (ASM), synthetic cystic fibrosis media (SCFM) and their derivatives ^19^^–^^21^ capture important chemical aspects of the CF airway environment, including extracellular DNA (eDNA), amino acids and mucins. ^20^^,^^21^ These media support infection-relevant biofilm formation and have been of significant value to the research community. Furthermore, they can be combined with host tissue to provide a physical scaffold for biofilm formation. ^22^ However, these models still lack key molecules of the lung, including host-derived antimicrobials, ^23^^,^^24^ polyamines ^25^^,^^26^ and a number of factors associated with CF comorbidities, including glucose, which is elevated in lungs in CF-related diabetes compared to healthy individuals, ^27^ and bile salts, which can be inhaled into lungs during CF-associated gastro-oesophageal reflux. ^28^

Most currently available *in vitro* and *in vivo* models focus on capturing conditions of the lung environment and therefore do not reflect the heterogeneity of the airways and are unsuitable for studying early infection processes that may take place in the sinuses. ^21^^,^^29^ Whilst *in vivo* infection models – most often performed in mice or rats - allow for a more holistic view of infection processes, ^30^ they are costly, hard to maintain and there are important differences in host-derived antimicrobial structure and function between rodents and man. ^31^ Furthermore, CF rodent models don’t reproduce the disease phenotypes and severity of human CF. ^30^ In particular, they poorly replicate the severe pathology of CF lung disease, with the major disease phenotype instead manifesting in the gastrointestinal tract. ^32^ The generation of gut-corrected CFTR transgenic mice has addressed this issue, but establishing chronic lung infection in these mice remains a challenge. ^32^ Embedding bacteria within agarose beads and instilling these into the trachea of mice allows for establishment of long-term infection, ^33^ albeit by an artificial route that requires surgical procedures and which is hard to standardise. Natural inhalation of CF-adapted *P. aeruginosa* suspended in saline offers an alternative that recapitulates the sinus to lung infection route and allows for long-term infection studies, ^8^ but this model has other drawbacks, including a relatively low density of infection in lungs. Study of major pathophysiological CF characteristics, such as chronic respiratory infection, inflammation, and mucus plugging, have been hampered by the limitations of existing *in vivo* models. ^32^

According to Home Office Report from 2021, 3.06 million procedures were performed involving living animals for scientific purposes. ^34^ More than half of these procedures were carried out in mice (54%) and 51% of overall procedures were for basic research while 27% was for applied research including human infectious disorders. ^34^ Moreover, in the field of respiratory microbiology, 10,540 publications report the use of mice for infection (Pubmed search “respiratory infection mouse” 2015-2022), more than half of these being for bacterial infections (6,274). Development of well-characterised respiratory mimicking media would offer opportunities for both replacement and reduction in such studies.

With the aim of understanding the applicability of available *in vivo* and *in vitro* models to study human infections, Cornforth et al developed a computational framework that uses transcriptomic data from *P. aeruginosa* grown under different environmental conditions and benchmarks it against the gene expression patterns observed in clinical CF sputum samples. This approach has been used to assess the clinical relevance of *in vitro* and *in vivo* models, with results suggesting that *in vitro* models are as good as or better than mouse lung infection models at mimicking the *P. aeruginosa* transcriptome in human CF sputum. ^35^

Given the limitations of the available animal models for study of CF infection and bacterial within-host adaptation, we sought to develop novel culture media that mimics the conditions of compartmentalised airway regions. To enable study of early infection processes taking place in the upper airways, we designed sinus-mimicking media reflective of conditions in health or in CF (healthy sinus media [HSM] and CF sinus media [CFSM]). In parallel, we developed media reflective of the lungs in health or CF (healthy lung media [HLM] and CF lung media [CFLM]). The developed CF media will be of use for the study of CF pathogens under infection-relevant conditions, whilst the healthy media equivalents can be used for study of airway infection in other contexts, such as non-CF bronchiectasis, community-acquired or ventilator-acquired pneumonia, that occur without the underlying altered environment that is specific to CF. Many of the differences between airway niches and between health and CF that we aim to capture have been described previously, including in O _2_/CO _2_ levels, temperature, ^36^ lysozyme, ^23^ lactoferrin, ^23^ polyamines, ^25^^,^^26^ mucins, ^37^ amino acids, carbon source content ^21^ and salt concentrations. ^38^ These media were designed to be cheap, accessible and readily modifiable, according to the research question being asked. Development of validated culture media that are more relevant than using nutrient broth and more cost effective and ethical than animal models can offer a platform to understand bacterial within-host adaptation, to use for isolate virulence or antimicrobial susceptibility testing, for efficacy testing of new therapeutics, or for study of bacterial growth, gene expression and biofilm formation under relevant environmental conditions.

## Methods

### Method for media development

**Microbial strains, base media and culture conditions**

All *P. aeruginosa* isolates used in this work are shown in Table 1. Bacteria were routinely streaked onto Tryptone Soy Agar (TSA) (Sigma-Aldrich) from bead stocks and restreaked every other day to maintain a fresh stock or from bead stocks, as required. After 18 hours of growth on agar, overnight cultures were prepared in 5ml Lysogeny Broth (LB) (Neogene) at 37°C and incubated for 18 hrs at 180 rpm. M9 medium (1 litre) was prepared by mixing 700 ml M9 salts (15 g/l KH _2_PO _4_, 64 g/l Na _2_HPO _4_, 2.5 g/l NaCl, 5.0 g/l NH _4_Cl) (Sigma-Aldrich) (autoclaved), 2 ml MgSO _4_ (autoclaved) (Sigma-Aldrich) and 20 ml 20% glycerol (Sigma-Aldrich) (filter-sterilized), in order. Sterilized water was then used to make up the volume to 1 L.

**Table 1.** Bacterial strains used in this study.

Strain	Description	Origin
PAO1	Widely used laboratory reference strain	Spontaneous chloramphenicol-resistant mutant of original PAO strain isolated from a wound in Melbourne, Australia ^63^
LESB65	Liverpool Epidemic Strain B65	CF chronic infection isolate of a transmissible strain from United Kingdom ^64^

**Single chemical growth rate assays**

**Figure 1.**
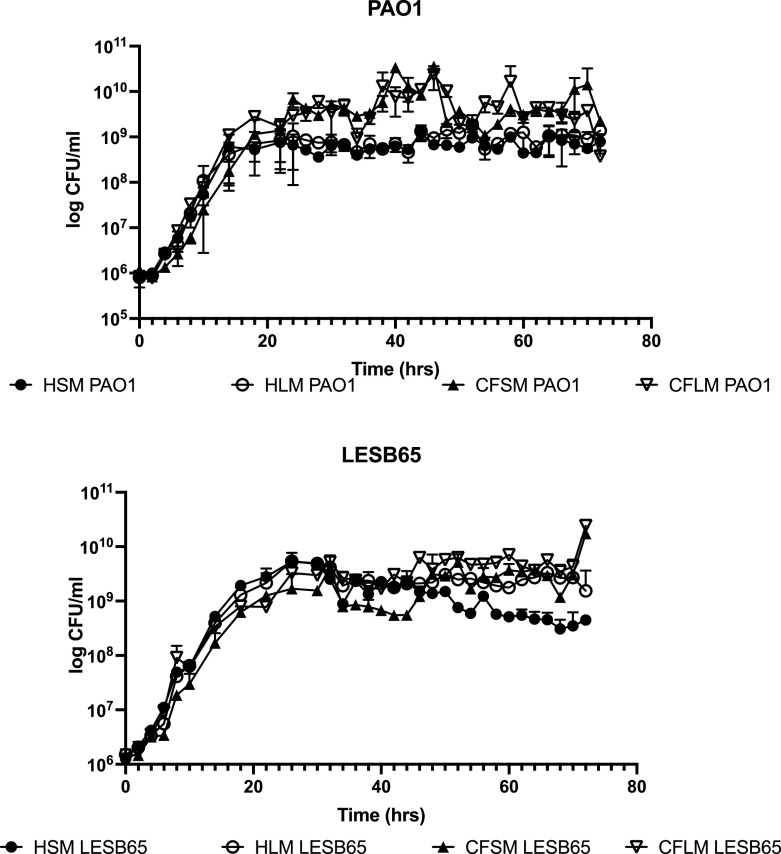
Continuous growth of PAO1 and LESB65 in respiratory media for 72 hrs. Media were generated using M9 supplemented with the ideal concentrations of all the chemicals shown in tables in media preparation protocol section. These concentrations differed between sinuses and lung and between health and CF, generating four different media: Healthy sinus media (HSM), CF sinus media (CFSM), healthy lung media (HLM) and CF lung media (CFLM). CFU counts are made by taking a sample from bacterial culture grown in each developed media, every 2 hours, for 72 hours. Data shows mean of 3 biological replicates per time point, error bars show standard deviation.

**Figure 2.**
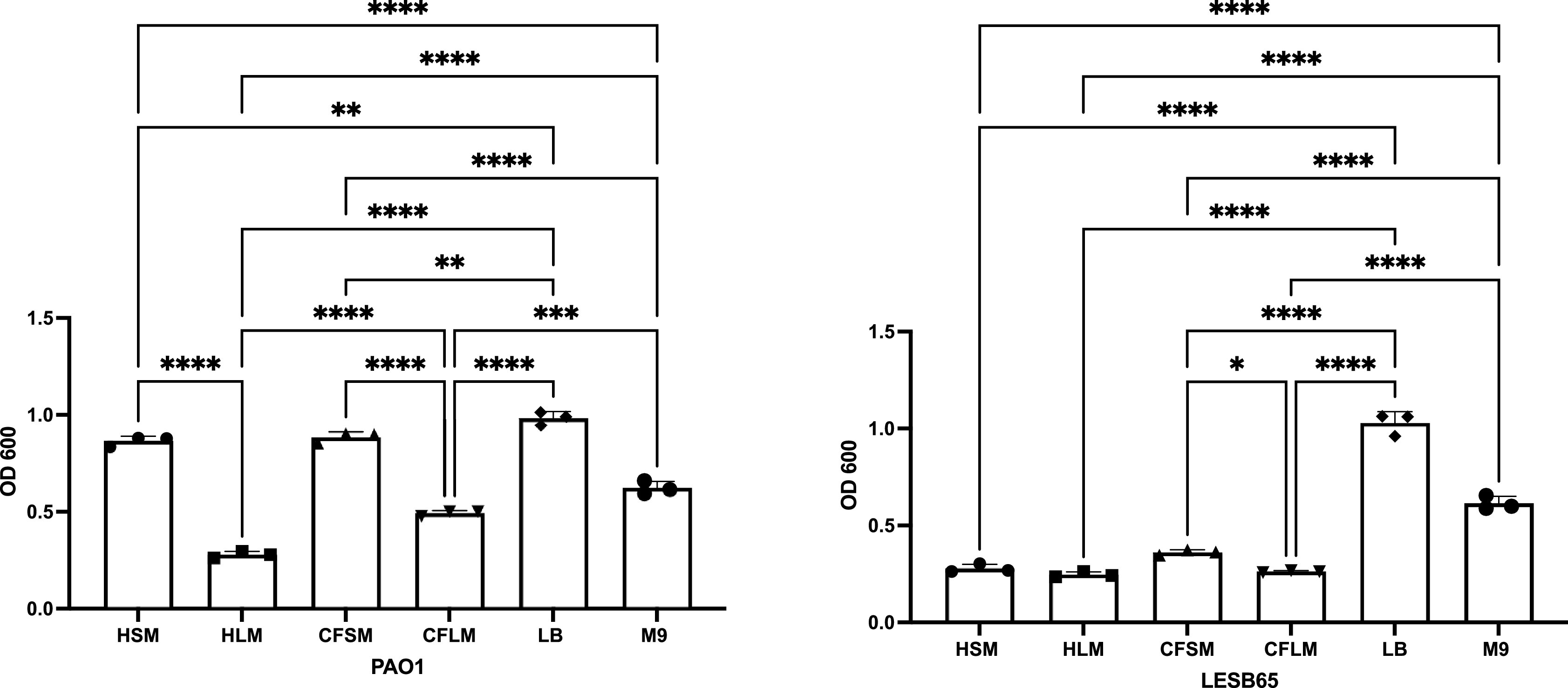
Attached biofilm biomass of PAO1 and LESB65 in respiratory-mimicking media, LB and M9. Bacteria were left to form biofilms for 72 hours. Unattached bacteria were removed and washed with PBS to ensure removal of all unattached bacteria. Attached bacteria stained with 0.25% CV and absorbances were measured by using 95-100% ethanol as blank control at OD600. Statistical analysis is made using Graph pad Prism 8.0. Statistical analysis: One-way ANOVA *p<0.05, **p<0.01, ***p<0.001, ****p<0.0001. Each bar graph represents 3 biological replicates (mean), each with 30 technical replicates, n:90, error bars show standard deviation).

**Figure 3.**
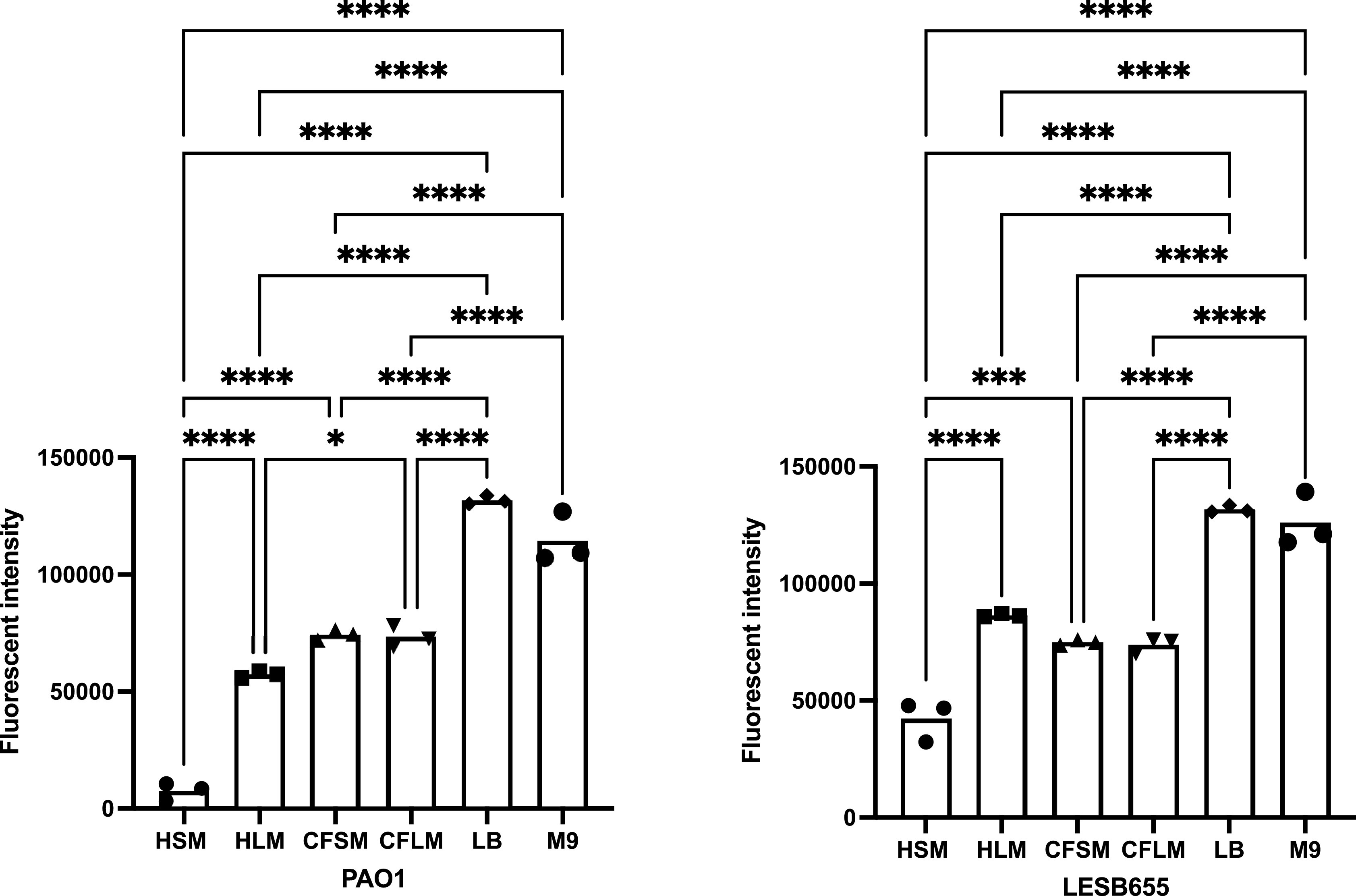
Free-floating biofilm formation in respiratory-mimicking media in comparison to two common laboratory media. Bacteria were left to form biofilms for 72 hours and biofilms were disrupted with cellulase. Cultures were incubated with resazurin dye for 2 hrs. Released fluorescence was measured at 540 nm excitation λ and 590 nm emission λ. Background fluorescence of each media is corrected by subtraction of the background fluorescence obtained from the wells with media only. Statistical analysis is made using Graph pad Prism 8.0. Statistical analysis: One-way ANOVA *p<0.05, **p<0.01, ***p<0.001, ****p<0.0001. Each bar graph represents 3 biological replicates (mean), each with 30 technical replicates, n:90, error bars show standard deviation).

**Figure 4.**
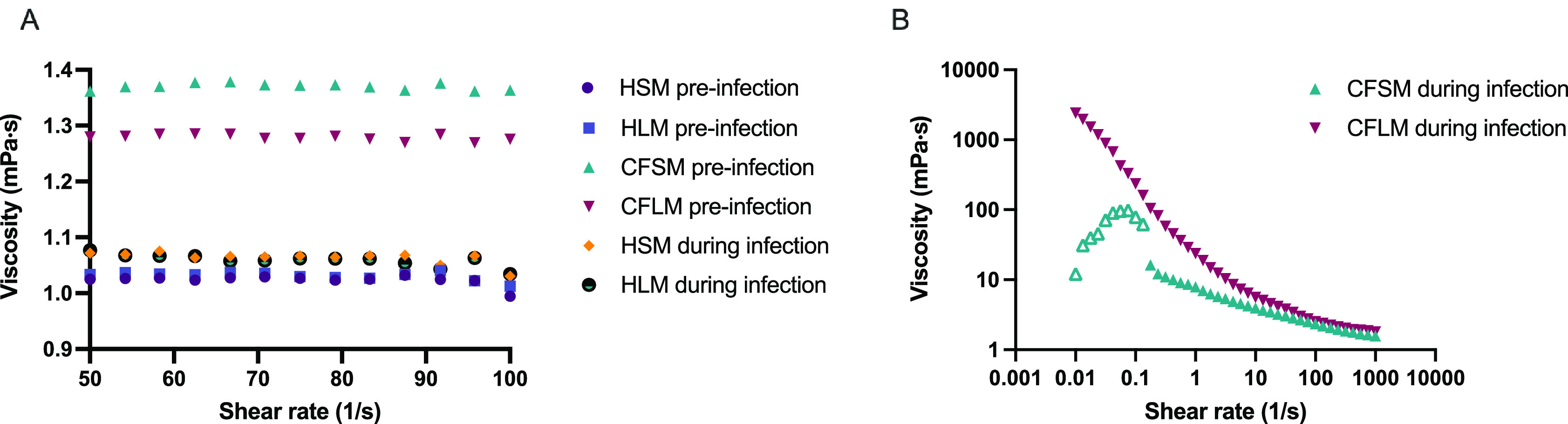
Viscosity of media before and after bacterial growth. Viscosity of all media before infection and healthy media during infection (A) was measured under shear rate range of 50-100 1/s whereas viscosity of CFSM and CFLM media was measured at 0.01-1000 1/s shear rate range as the viscosity properties of CF media during infection was higher (B). Averages were taken for media with Newtonian fluid characteristics (All media before infection and HSM and HLM media during infection). Data points with open triangle in CFSM during infection displayed high level of uncertainty due to non-Newtonian responses and therefore values below 0.1 (1/s) should be treated with caution. Graphs are showing single viscosity measurement per media under each condition.

Each chemical under consideration for inclusion in the respiratory tract-mimicking media was first assessed by supplementation individually into M9 media at ‘low’, ‘ideal’ or ‘high’ concentrations. Ideal concentrations represent those estimated to most closely reflect the physiological concentration found in each niche, in either health or disease. The relevance of the final list of chemicals included in the final media, in the context of bacterial adaptation to airway environments, is summarised in Table 2. The tested concentration ranges were determined by reference to the literature or from experimental measurement of metabolites ( Table 3). Low and high concentration values were set at 2-fold below and above the ideal concentration of each chemical. Growth rates of PAO1 and LESB65 were assessed in M9 media supplemented with each chemical, using a microplate reader (Varioskan ^®^ LUX, Thermo Scientific) (Underlying data Figures 1-4). 2 μl of bacteria from overnight cultures were added to 198 μl of M9 media supplemented with the appropriate chemical. The bacteria were then incubated in sterile 96-well plates (U-bottom) (Greiner) for 20 hours and the growth rates assessed at OD _600nm_ at 10 minutes intervals. Incubation conditions were 37°C/5% CO _2_ for lung conditions and 34°C/0% CO _2_ for sinus conditions. Three biological replicates were performed, per bacterial isolate, per condition. A single working concentration to be used in the final pooled media was then chosen for each chemical, per condition and growth rate assays were performed again with the chosen concentration before the chemicals were combined into a single pooled media. The ‘ideal’ concentration was chosen for the final media unless found to cause complete inhibition of bacterial growth.

**Table 2.** Chemicals included in the media and their importance in shaping the bacterial adaptation in airways.

Chemicals	Importance in the airways
Host-derived antimicrobials • Lysozyme • Lactoferrin	• The effectiveness of lysozyme reduces in biofilms and in the chronically infected lung, due to electrostatic sequestration of the enzyme by infection-associated anionic biopolymers ^33^^,^^34^ • *Pseudomonas aeruginosa* acquire mutations leading to resistance against lysosomal killing in the lung. ^10^ • In CF, activity of lactoferrin can be inhibited by causing partial cleavage via high concentration of cathepsin (neutrophil elastase) and Pseudomonas elastase secretions. ^65^
Polyamines • Spermidine • Spermine • Putrescine	• *P. aeruginosa* utilizes host-derived polyamines to facilitate antimicrobial tolerance. ^25^ • Spermidine was shown to readily coat the surface of PmrB *-*deficient *P. aeruginosa*, protecting *P.aeruginosa* from antibiotics and oxidative stress and leading to increase in biofilm formation. ^25^
Mucin	• The abnormal mucin glycosylation in CF promotes bacterial adhesion and infection, via increased exposure of specific glycan receptors. ^66^ • *P. aeruginosa* can break down and utilize mucin glycans as monosaccharides. ^67^
eDNA	• Necrosis products of neutrophils and component of extracellular polymeric substance (EPS) in biofilms. • Presence of neutrophils in infection leads to increased biofilm formation as result of formation of eDNA-actin polymers. ^68^ • Bacterial eDNA can also be secreted by *P. aeruginosa* to aid biofilm formation. ^69^ • eDNA can shield *P. aeruginosa* biofilms against aminoglycosides and protect them from antimicrobial attack. ^69^ • Presence of eDNA leads to formation of cation gradients and inducible antibiotic resistance in CF airways. ^70^
Amino acids	• Bacteria exoproducts or derived from the host. • *P. aeruginosa* secretes exoproteases to generate more utilisable nutritional substrates to support its growth. ^71^ • Increased amino-acid content in the airways of CF patients plays a significant role in the selection and maintenance of nutritionally deficient *P. aeruginosa.* ^71^ • In the presence of amino acids, *P. aeruginosa* quorum sensing activity and exopolysaccharide formation increases. ^72^
Serum Albumin	• Binding of albumin can promote bacterial survival at the epithelial cell surface. ^53^ • Albumin influences formation of *P. aeruginosa* virulence factors. Serum albumin shown to play a critical role in *P. aeruginosa* virulence during early phases of infection by enhancing the expression of iron-controlled genes. ^73^
Biometals • Calcium Chloride (CaCl _2_) • Magnesium Chloride (MgCl _2_) • Iron Chloride (FeCl _2_) • Copper Chloride (CuCl _2_) • Zinc Chloride (ZnCl _2_)	• Iron and zinc aid bacterial growth and are essential nutrients for bacteria ^56^ • Magnesium has been proposed to have a role in maintaining established biofilms ^56^
Carbohydrate sources • Sialic acid • Galactose • N-acetyl glucosamine • Glucose	• Products of mucin degradation in CF airways, act as nutrient sources. ^54^ • Glucose elevated in lungs in CF-associated diabetes. ^40^
Salts • Sodium Chloride • Bile salts • Succinate	• High concentrations of sodium chloride in the airway surface liquid (ASL) inhibit the activity of antimicrobial factors giving higher chance for bacterial persistence. ^74^ • Long term bile exposure shown to cause emergence of adaptive *P. aeruginosa* variants that are ecologically competitive sub-populations. ^75^ • Succinate-exposed *P. aeruginosa* showed increase in glucose metabolism and utilization of trehalose and acetate, production of EPS and enabled tolerance to oxidant stress ^76^

**Table 3.** Concentration of each chemical to be used in the final media. Amino acid concentration sources: healthy ^77^: CF ^21^: (x: chemical not present).

Chemicals	Healthy sinuses	CF sinuses	Healthy lungs	CF lungs
Lysozyme	8 μg/ml ^23^	24 μg/ml ^23^	8 μg/ml ^23^	48 μg/ml ^23^
Lactoferrin	0.5 μg/ml ^23^	50 μg/ml ^23^	0.5 μg/ml ^23^	50 μg/ml ^23^
Spermidine	200 ng/ml ^25^	325 ng/ml ^25^	250 ng/ml ^25^	350 ng/ml ^25^
Spermine	32.5 μg/l ^26^	346 μg/l ^26^	44.5 μg/l ^26^	346 μg/l ^26^
Putrescine	616 μg/l ^26^	616 μg/l ^26^	616 μg/l ^26^	616 μg/l ^26^
Mucin	1.2 mg/ml ^37^	5 mg/ml ^29^	1.2 mg/ml ^37^	5 mg/ml ^29^
eDNA	0.96 mg/ml ^78^	4 mg/ml ^29^	0.96 mg/ml ^78^	4 mg/ml ^29^
Albumin	0.5 mg/ml ^54^	7 mg/ml ^54^	1.5 mg/ml ^54^	7 mg/ml ^54^
CaCl _2_	45 mg/l ^56^	100 mg/l ^56^	45 mg/l ^56^	100 mg/l ^56^
MgCl _2_	8 mg/l ^56^	30 mg/l ^56^	8 mg/l ^56^	30 mg/l ^56^
FeCl _2_	887 μg/l ^56^	5.95 mg/l ^56^	887 μg/l ^56^	5.95 mg/l ^56^
CuCl _2_	106 μg/l ^56^	173 μg/l ^56^	106 μg/l ^56^	173 μg/l ^56^
ZnCl _2_	390 μg/l ^56^	1285 μg/l ^56^	390 μg/l ^56^	1285 μg/l ^56^
NaCl	1 mg/ml ^38^	6.3 mg/ml ^38^	1 mg/ml ^38^	6.3 mg/ml ^38^
Sialic acid	3.23 μg/l ^79^	6.46 μg/l ^79^	3.23 μg/l ^79^	6.46 μg/l ^79^
Bile	X	X	X	0.3 mg/ml ^80^
N-acetyl glucosamine	1.28 mg/ml ^54^	4.42 mg/ml ^54^	1.28 mg/ml ^54^	4.42 mg/ml ^54^
Glucose	X baseline glucose (20%) from M9 media components	250 mg/l ^40^	X baseline glucose (20%) from M9 media components	250 mg/l ^40^
Succinate	0.295 mg/ml ^76^	2.95 mg/ml ^76^	0.295 mg/ml ^76^	2.95 mg/ml ^76^
Galactose	6.27 μg/l ^79^	7 μg/l ^79^ ^79^	6.27 μg/l ^79^	7 μg/l ^79^
L-Methionine	0.3 mg/l	90 mg/l	0.3 mg/l	90 mg/l
L-Phenylalanine	0.8 mg/l	90 mg/l	0.8 mg/l	90 mg/l
L-Proline	1.3 mg/l	200 mg/l	1.3 mg/l	200 mg/l
L-Serine	1.8 mg/l	150 mg/l	1.8 mg/l	150 mg/l
L-Threonine	15 mg/l	120 mg/l	15 mg/l	120 mg/l
L-Tryptophan	0.16 mg/l	2 mg/l	0.16 mg/l	2 mg/l
L-Valine	1.6 mg/l	130 mg/l	1.6 mg/l	130 mg/l
L-Ornithine	X	90 mg/l	X	90 mg/l
L-Tyrosine	0.73 mg/l	140 mg/L	0.73 mg/l	140 mg/L
L(+)-Asparagine monohydrate	0.35 mg/l	X	0.35 mg/l	X
L-Alanine	3 mg/l	160 mg/l	3 mg/l	160 mg/l
L-Arginine	37 mg/l	50 mg/l	37 mg/l	50 mg/l
L(+)-Aspartic acid	1 mg/l	110 mg/l	1 mg/l	110 mg/l
L-Cysteine	0.28 mg/l	24.2 mg/l	0.28 mg/l	24.2 mg/l
L-Glutamine	0.5 mg/l	220 mg/l	0.5 mg/l	220 mg/l
L-Glycine	2.7 mg/l	90 mg/l	2.7 mg/l	90 mg/l
L-Histidine	0.5 mg/l	77.5 mg/l	0.5 mg/l	77.5 mg/l
L-Isoleucine	1 mg/l	140 mg/l	1 mg/l	140 mg/l
L-Leucine	2 mg/l	210 mg/l	2 mg/l	210 mg/l
L-Lysine	2.2 mg/l	310 mg/l	2.2 mg/l	310 mg/l

### Preparation of the pooled media

Media synonyms:

Healthy sinus media (HSM),

Healthy lung media (HLM),

CF sinus media (CFSM),

CF lung media (CFLM).

Media components were pooled together at concentrations given in Table 3, yielding four different respiratory media: Healthy sinus media (HSM), healthy lung media (HLM), CF sinus media (CFSM) and CF lung media (CFLM). Briefly, eDNA and mucin solutions were prepared separately in distilled water by constant stirring overnight at 4 °C. Next day, the solutions were autoclaved at 121 °C. Other components of the media were prepared in a single solution by firstly adding the M9 media components (water, 20% glucose and 1M MgSO _4_). Stock solutions of all the media components except glucose, succinate, N-acetyl glucosamine (Glc-NAc), bile, NaCl, albumin and amino acids were prepared and added to the media in solution form. Aforementioned chemicals were then added in powder form, slowly, under constant stirring at room temperature. Once all the components were fully mixed, the solution was sterilized by filtration using Vacuubrand ME 2 diaphragm vacuum pump and Millipore Steritop filter units with a pore and neck size of 0.22 μm. Sterile eDNA and mucin was then added to the filtered media and the pH of media was adjusted to 6.8-6.9 using sodium hydroxide (NaOH) (Sigma-Aldrich). The volume of the solution was brought to 1 litre by the addition of autoclaved distilled water. The media was then aliquoted to 50ml falcon tubes and stored at -80°C until further use.

### Salinity adjustments

M9 media was prepared without the addition of M9 salts because exogenous salts were added in the form of NaCl and bile salts, as defined in Table 3. Salinity of the media was adjusted according to the salinity of ASM (2%) (measured in this study) for CF media. Salinity of healthy media was kept lower than CF media. Briefly, 1ml of media (ASM, HSM, HLM, CFSM, CFLM) was deposited to the surface of refractometer (V-RESOURCING) after it was calibrated with sterile water. Salinity of CF media was adjusted to 2%, salinity of healthy media was kept less than 2% as CF airways is known to have higher salt content than healthy airways, although salinity in health is not well defined.

### Viscosity measurements

Viscosity of media was measured before and after allowing bacteria to form biofilms for three days, to mimic chronic infection. Viscosity measurements (mPa.s) were performed using a rheometer (Anton-Paar) with cone plate (Serial number: 44806). 50-100 1/s range was used for all sterile media and healthy media during infection, and 0.01-1000 1/s range was used for CF media during infection. Changes in media viscosity was observed under each shear rate point.

### Media stability

Stability of developed media was tested over a 30-day period by performing crystal violet stain assays (as described below) using media stored at 4°C for 30 days. This experiment was designed to determine whether media would continue to support biofilm formation after prolonged storage.

### Growth curves of PAO1 and LESB65 in the developed media

Colony forming unit (CFU) assays were performed to observe the growth of PAO1 and LESB65 in the pooled media. Bacterial cultures grown in LB for 18 hours were centrifuged for 12 minutes at 3220rcf at room temperature. After the LB suspension was removed, bacterial pellets were resuspended in 5 ml of sterile phosphate buffer saline (PBS) (Sigma-Aldrich). The suspension was homogenized by vortexing and bacterial optical density was adjusted to OD 0.05-0.06 in PBS using an optical spectrometer (HANNA instruments). 50 ul of this suspension was then added to 4950 ul HSM, HLM, CFSM or CFLM and cultures were incubated under niche-appropriate conditions: sinus media at 34°C with no CO2, lung media at 37°C with 5% CO _2_. Anaerobic jars were used to increase the CO _2_ concentration for lung conditions. CFU was measured at pre-determined time intervals by serial-dilution onto agar. Briefly, 10-fold serial dilutions were prepared in 96 well microplates (Greiner U-bottom) using PBS as the diluent. 20 ul of bacterial dilution was then plated to TSA plates covering the dilutions from 10 ^-2^ to 10 ^-9^. The plates were air dried and then incubated at 37°C until the colonies were clearly visible (18 hours for PAO1 and 24 hours for LESB65).

### Biofilm formation in the developed media

**Measurement of attached biofilm biomass by crystal violet staining**

Starting from an overnight liquid culture in LB (incubated for 18 hours), bacterial cultures were diluted 1:100 in the developed media, or in LB, or M9, two widely used laboratory bacterial culture media. To minimise edge-effect, non-perimeter wells of U-bottomed polystyrene 96-well microtiter plate (Greiner U-bottomed) were inoculated with 180 μl of these dilutions and perimeter wells were used as a negative control (sterile medium). Following 3 days of growth at 37°C, the supernatant (containing non-adhered cells) was removed from each well and plates were rinsed using 200 μl sterile PBS, twice. Subsequently, 200 μl of Crystal Violet (CV) was added to non-perimeter wells and plates were incubated at room temperature for minimum 20 minutes. After 20 minutes, the excess CV was removed by washing the plates under running tap water. Finally, bound CV was released by addition of 200 μl of 95-100% ethanol (Sigma-Aldrich) to the wells with bacteria. After incubating with ethanol for 30 minutes, the absorbance was measured at 600 nm using a BMG plate reader. 95-100% ethanol was used as a blank.

**Measurement of cell viability and free-floating biofilm formation by resazurin assays**

Overnight cultures of PAO1 and LESB65 were diluted in LB to an OD 600 of 0.05 (±0.01). These cultures were then further diluted in the developed media (1:100) to a total volume of 10ml in glass universal tubes. Free floating biofilm formation in respiratory tract-mimicking media was compared to that observed in LB and M9. Diluted developed media cultures were incubated under conditions appropriate for each respiratory niche, as described above. Cultures were incubated for 3 days while shaking at 75 rpm. Three glass universal tubes containing bacteria-free respiratory media, LB or M9 were used as a negative control. After the incubation, biofilms were disrupted using 250 μl of 100 mg/ml cellulase (diluted in 0.05 M citrate buffer [9.6 g/l Citrate.H _2_O (VWR)] in water and pH to 4.6 with NaOH) and further incubated under aerobic conditions at 37 °C, while shaking at 150 rpm for 1 h. After 30 minutes incubation, biofilms were further disrupted by manual pipetting to ensure complete disruption of biofilms. A portion of disrupted biofilms were then transferred to 96-well plates (Greiner U-bottomed) and to determine the metabolic activity of the bacterial cells released from the disrupted biofilms, 10 μl of 0.02 % (v/v) resazurin (Sigma-Aldrich) (diluted in distilled water) was added to each well of the 96-well plates and incubated for 1-2 hours at niche specific temperature, while shaking at 150 rpm. Following incubation with resazurin, the fluorescence of each well was measured using an excitation wavelength of 540 nm and an emission wavelength of 590 nm in a Fluostar Omega microplate reader and the MARS Data Analysis Software.

### RNA extraction and cDNA synthesis

The bacteria were grown in 5ml of each developed media or LB until early stationary phase (12 hrs for PAO1 and 20 hrs for LESB65 in the developed media, and 6hrs for PAO1 and 18hrs for LESB65 in LB). To extract RNA, TRI reagent (ZYMO Research) was added and mixtures were incubated for 5–10 min at room temperature. Bacteria were harvested by centrifugation for 30 min at 13,000 rpm and 4 °C. Supernatant was removed and bacterial pellets were used for subsequent RNA isolation or stored at −80 °C. RNA from bacterial cultures was isolated using the Direct Zol RNA Microprep kit (ZYMO Research: R2061) according to the manufacturer’s instructions. DNase digestion treatment was performed using DNAse 1 (ZYMO Research) as per the manufacturer’s protocol. Isolation was performed in an RNase-free environment using RNase-free consumables and reagents. Purified RNA was eluted with RNase-free water (ZYMO Research). Quantification of RNA was performed by measuring the absorbance at 260 nm wavelength using the NanoDrop8000 UV–vis Spectrophotometer (Thermo Scientific). and purity of RNA was analysed by determination of the 260/280 nm ratio. Nuclease free water (Invitrogen) was used as a blank. The 260/280 nm ratio obtained for all samples was between 1.8 and 2.0.

First-strand cDNA synthesis was performed using the iScript cDNA synthesis kit (BIO-RAD: 1708891) according to the manufacturer’s instructions in an RNase-free environment using RNase-free consumables and reagents. For each sample, 2.5 ng RNA was added and incubated in a thermocycler (Applied Biosystem) under the following protocol: 5 min at 25 °C, 30 min at 42 °C and then 5 min at 85 °C. The cDNA was then stored at −20 °C until further use.

### Quantitative real-time PCR (qPCR) and gene expression analysis

cDNA was used for quantitative real-time PCR (qPCR) in duplicate reactions using the GoTaq ^®^ qPCR Master Mix (Promega: A6001) as per the manufacturer’s instructions. Each reaction contained 2 μl of cDNA (or nuclease free water for the no template control) and 0.2 μM of forward and reverse primers (Eurofins). Primer sequences are provided in Table 4. qPCR was performed using the BioRad CFX Connect Real Time PCR System (BIORAD) using MicroAmp™ Optical 96-Well Reaction Plate (Applied Biosystem) under the following conditions; 2 min at 95 °C followed by 40 cycles of 15 sec at 95 °C and 1 min at 60 °C.

**Table 4.** Primer sequences used in the qPCR experiments for PAO1 and LESB65.

**PAO1**	
algU Forward primer	CAGGAACAGGATCAGCAACT
algU Reverse primer	CGCACGATCAATCCCAGTAT
mexB Forward primer	GTTCCTGGTGATGTACCTGTT
mexB Reverse primer	GTTCCTGGTGATGTACCTGTT
PA2911 Forward primer	AGCAACAACTCGCCCTATAC
PA2911 Reverse primer	CGAGCGGCTTTCGAAGTA
PA2382 Forward primer	TACACCCTGTCGACCATGA
PA2382 Reverse primer	GCATCACGTAGAGCTGGAAC
rpoD Forward primer	GGGCGAAGAAAGGAAATGGT
rpoD Reverse primer	CAGGTGGCGTAGGTGCAGA
**LESB65**	
algU Forward primer	CAGGAACAGGATCAGCAACT
algU Reverse primer	CGCACGATCAATCCCAGTAT
mexB Forward primer	CGATCCATGAGGTAGTGAAGAC
mexB Reverse primer	GTTCTGCAGGAACAGGTACA
PA2911 Forward primer	AGCAACAACTCGCCCTATAC
PA2911 Reverse primer	CGAGCGGCTTTCGAAGTA
PA2382 Forward primer	CCGTTCCTCTTCCACTACATC
PA2382 Reverse primer	TCAGCTCGGACATGTTCTTC
rpoD Forward primer	Same rpoD primers with PAO1
rpoD Reverse primer	Same rpoD primers with PAO1

Each qPCR was performed with three biological replicates and in duplicate on each run. Gene *rpoD*, encoding sigma factor RpoD, was used as a reference gene. cDNA obtained from cultures grown in LB was used as a control. Analysis of relative gene expression in each developed media and SCFM2 versus (vs) LB was performed using the 2 ^−ΔΔCt^ relative quantification method as described previously. ^39^

### Long term culturing of PAO1 in developed media

PAO1 was cultured in the developed media for forty days, sub-cultured to fresh media every 2 days and plated on Tryptone Congo red/Coomassie blue Agar (TCCA) prepared by mixing 20 mg/L Coomassie blue (Sigma-Aldrich), 40 mg/L Congo red (Sigma-Aldrich), 10 g/L Tryptone (Sigma-Aldrich) and 12 g/L Bacto agar (Fisher-Scientific) in distilled water and autoclaving at 121°C for 15 minutes. 100 μl of bacterial cultures were spread on to the plates in serial dilutions (10 ^-4^, 10 ^-6^) every ten days. Plates were incubated at 37°C for 24 hours and for a further 48 hours at room temperature to allow the colonies to uptake the two dyes in the media.

### Statistical analysis

All assays were carried out at least three times independently. Statistical significance was evaluated using One-way ANOVA (*p<0.05, **p<0.01, ***p<0.001, **** p<0.0001). All statistical analysis was done using Graphpad Prism versions 8 and 9 (similar analysis could be performed in Microsoft Excel as a free to use alternative). For single chemical growth rate assays, R studio version 3.6.2 and GrowthCurver (version 0.3.1) was used to plot the area under the curve (AUC_e) values.

### Protocol for media preparation

Here we describe, step-by step, the procedure used to prepare 1L of each of the four developed media. Reagents and equipment used in this study are listed in Table 16.

**Preparation of the base (same for both CF media)**

1. In a 1 litre Duran bottle, add 300 ml distilled water, 20 ml 20% glucose and 2 ml 1 M MgSO _4_.

**Preparation of CF sinus media**

1. Add 4.8 g eDNA in 300 ml water under continuous stirring with a magnetic stirrer at room temperature. Add the eDNA very slowly as it can form clumps in the water.
2. Add 6 g mucin from porcine stomach (Type II) in 150 ml water under continuous stirring with a magnetic stirrer at room temperature. Add mucin powder slowly to water to prevent the formation of clumps. eDNA and mucin can be stirred overnight at 4°C to fully dissolve the powder.
3. Once the eDNA and mucin solutions are fully dissolved, autoclave at 121°C for 15 minutes to sterilise.
4. Prepare stocks of lysozyme, lactoferrin, spermidine, spermine, putrescine, CaCl _2_, MgCl _2_, FeCl _2_, CuCl _2_, ZnCl _2_, sialic acid and galactose in the concentrations given in Table 5 for CF sinuses by dissolving each powder in deionised water.
5. Under constant stirring with magnetic stirrer, from the stocks prepared, add the amounts of each chemical to the base media as given in Table 6.
6. Add 35 μl of neat spermidine solution (9.296 g/l) to the base media.
7. Weigh the amounts of L-amino acids given in Table 3 and add one by one to the media under constant stirring. Exceptions are L-cysteine and L-tyrosine. Dissolve L-cysteine in 2.95 ml of 0.5 M potassium hydroxide (M _r_ 56.11 g/mol) and L-tyrosine in 2.95 ml sterile water, separately before adding them to the base media. (Amino acid concentrations are same for both CF sinuses and CF lung media.)
8. Weigh 7 g of albumin, 6.3 g of NaCl, 4.4 g of N-acetyl glucosamine, 250 mg of glucose and 2.95 g of succinate and add to the media one at a time. Wait for each chemical to dissolve completely before adding the next one. Succinate can take time to dissolve in water. Incubate the succinate at 37 °C while shaking at 180 rpm for 10-15 minutes to quicken dissolving.
9. Sterilise the media by filtration using a Vacuubrand ME 2 diaphragm vacuum pump and Millipore Steritop filter units with a pore and neck size of 0.22 μm.
10. Under sterile conditions, add 250 ml of autoclaved eDNA and 125 ml of autoclaved mucin to the mixture.
11. Adjust pH to 6.8-6.9 with 5 M NaOH and bring the volume to 1 litre with sterile water.
12. Filtered media should be stored at 4 °C for further use. Using fresh media is recommended, however it can be kept under these conditions for a maximum of three weeks. For freshness, the media can be aliquoted and stored at -80°C (suitable for longer term use).

**Table 5.** Preparation of chemical stocks for CF sinuses media.

Chemical stock preparation for CF sinuses media			
Chemical	Stock concentration (mg/ml)	Amount of powder to weigh to form the stock solution (mg)	Volume of water to dissolve the powder in (ml)
Lysozyme	1	24	24
Lactoferrin	1	50	50
Spermine	1	1	1
Putrescine	200	200	1
CaCl _2_	250	250	1
MgCl _2_	100	100	1
FeCl _2_	1	6	6
CuCl _2_	1	1	1
ZnCl _2_	2.6	2.6	1
Sialic acid	1	1	1
Galactose	1	1	1

**Table 6.** Volume of each chemical to add into the base media for 1liter CFSLM.

CF sinuses media			
Chemical	Stock concentration	Concentration desired in the media	Amount to add from the stock
Lysozyme	1	24 μg/ml	24 ml
Lactoferrin	1	50 μg/ml	50 ml
Spermine	1	346 μg/l	346 μl
Putrescine	200	616 μg/l	3 μl
CaCl _2_	250	100 mg/l	400 μl
MgCl _2_	100	30 mg/l	300 μl
FeCl _2_	1	5.95 mg/l	5.95 ml
CuCl _2_	1	173 μg/l	173 μl
ZnCl _2_	2.6	1285 μg/l	494 μl
Sialic acid	1	6.46 μg/l	6.46 μl
Galactose	1	7 μg/l	7 μl

**Preparation of CF lung media**

1. Follow steps 1-3 of CFSM protocol for eDNA and mucin stock solution preparation.
2. Prepare stocks of lysozyme, lactoferrin, spermidine, spermine, putrescine, CaCl _2_, MgCl _2_, FeCl _2_, CuCl _2_, ZnCl _2_, sialic acid and galactose in the concentrations given in Table 7 for CF lungs by dissolving each powder in deionised water.
3. Under constant stirring with magnetic stirrer, from the stocks prepared, add the amounts of each chemical to the base media as given in Table 8.
4. Add 37.6 μl of neat spermidine solution (9.296 g/L) to the base media.
5. Add amino acids in the same way as for preparation of CF sinuses media (See Table 3).
6. Add 7 g albumin, 6.3 g NaCl, 4.4 g of N-acetyl glucosamine, 250 mg of glucose, 2.95 g of succinate, 300 mg bile salts, one at a time.
7. Sterilise the media in the same way to CF sinuses media (see above protocol).
8. Under sterile conditions, add 250 ml of autoclaved eDNA and 125 ml of autoclaved mucin to the mixture.
9. Adjust pH to 6.8-6.9 with 5M NaOH and bring the volume to 1 litre with sterile water.
10. Filtered media should be stored at 4 °C for further use. Using fresh media is recommended, however it can be kept under these conditions for a maximum of three weeks. For freshness, the media can be aliquoted and stored at -80°C (suitable for longer term use).

**Table 7.** Preparation of chemical stocks for CF lungs media.

Chemical stock preparation for CF lungs media			
Chemical	Stock concentration (mg/ml)	Amount of powder to weigh to form the stock solution (mg)	Volume of water to dissolve the powder in (ml)
Lysozyme	1	48	48
Lactoferrin	1	50	50
Spermine	1	1	1
Putrescine	200	200	1
CaCl _2_	250	250	1
MgCl _2_	100	100	1
FeCl _2_	1	6	6
CuCl _2_	1	1	1
ZnCl _2_	2.6	2.6	1
Sialic acid	1	1	1
Galactose	1	1	1

**Table 8.** Volumes of each chemical to add into the base media for 1liter CFLM.

CF lungs media			
Chemical	Stock concentration (mg/ml)	Concentration desired in the media	Amount to add from the stock
Lysozyme	1	48 μg/ml	48 ml
Lactoferrin	1	50 μg/ml	50 ml
Spermine	1	346 μg/l	346 μl
Putrescine	200	616 μg/l	3 μl
CaCl _2_	250	100 mg/l	400 μl
MgCl _2_	100	30 mg/l	300 μl
FeCl _2_	1	5.95 mg/l	5.95 ml
CuCl _2_	1	173 μg/L	173 μl
ZnCl _2_	2.6	1285 μg/L	494 μl
Sialic acid	1	6.46 μg/L	6.4 μl
Galactose	1	7 μg/L	7 μl

**Healthy sinuses media**

**Preparation of the base media (Same for both healthy media)**

1. In a 1 litre Duran bottle, add 250 ml distilled water, 20 ml 20% glucose and 2 ml 1 M MgSO _4_.

**Preparation of healthy sinuses media**

1. Add 1 g eDNA in 500 ml water under continuous stirring with a magnetic stirrer at room temperature. Add the eDNA very slowly as it can form clumps in the water.
2. Add 1.4 g mucin from porcine stomach (Type II) in 140 ml water under continuous stirring with a magnetic stirrer at room temperature. Add mucin powder slowly to water to prevent the formation of clumps. eDNA and mucin can be stirred overnight at 4 °C to fully dissolve the powder.
3. Prepare stocks of lysozyme, lactoferrin, spermidine, spermine, putrescine, CaCl _2_, MgCl _2_, FeCl _2_, CuCl _2_, ZnCl _2_, sialic acid and galactose in the concentrations given in Table 9 for healthy sinuses by dissolving each powder in deionised water.
4. Under constant stirring with magnetic stirrer, from the stocks prepared, add the amounts of each chemical to the base media as given in Table 10.
5. Add 21.5 μl of neat spermidine solution (9.296 g/l) to the base media.
6. Add amino acids in the same way as for preparation of the CF media, see Table 3 *.*
7. Add 500 mg albumin, 1g NaCl, 1.28 g of N-acetyl glucosamine, one at a time.
8. Sterilise the media in the same way as for CF sinuses media (see above protocol)
9. Under sterile conditions, add 480 ml of autoclaved eDNA and 120 ml of autoclaved mucin to the mixture.
10. Adjust pH to 6.8-6.9 with 5 M NaOH and bring the volume to 1 litre with sterile water.
11. Filtered media should be stored at 4 °C for further use. Using fresh media is recommended, however it can be kept under these conditions for a maximum of three weeks. For freshness, the media can be aliquoted and stored at -80 °C (suitable for longer term use).

**Table 9.** Preparation of chemical stocks for healthy sinuses media.

Chemical stock preparation for healthy sinuses media			
Chemical	Stock concentration (mg/ml)	Amount of powder to weigh to form the stock solution (mg)	Volume of water to dissolve the powder in (ml)
Lysozyme	1	8	8
Lactoferrin	1	1	1
Spermine	1	1	1
Putrescine	200	200	1
CaCl _2_	250	250	1
MgCl _2_	100	100	1
FeCl _2_	1	5.95	5.95
CuCl _2_	1	1	1
ZnCl _2_	2.6	2.6	1
Sialic acid	1	1	1
Galactose	1	1	1
Succinate	80	320	4

**Table 10.** Volumes of each chemical to add into the base media for 1 litre HSLM.

Healthy sinuses media			
Chemical	Stock concentration (mg/ml)	Concentration desired in the media	Amount to add from the stock
Lysozyme	1	8 μg/ml	8 ml
Lactoferrin	1	0.5 μg/ml	500 μl
Spermine	1	32.5 μg/l	32.5 μl
Putrescine	200	616 μg/l	3 μl
CaCl _2_	250	45 mg/l	180 μl
MgCl _2_	100	8 mg/l	80 μl
FeCl _2_	1	887 μg/l	887 μl
CuCl _2_	1	106 μg/l	106 μl
ZnCl _2_	2.6	390 μg/l	150 μl
Sialic acid	1	3.23 μg/l	3.23 μl
Galactose	1	6.27 μg/l	6.27 μl
Succinate	80	0.295 mg/ml	3.6 ml

**Healthy lungs media**

**Preparation of healthy lungs media**

1. Follow steps 1-3 of HSM protocol for eDNA and mucin stock solution preparation.
2. Prepare stocks of lysozyme, lactoferrin, spermidine, spermine, putrescine, CaCl _2_, MgCl _2_, FeCl _2_, CuCl _2_, ZnCl _2_, sialic acid and galactose in the concentrations given in Table 11 for healthy lungs by dissolving each powder in deionised water.
3. Under constant stirring with magnetic stirrer, from the stocks prepared, add the amounts of each chemical to the base media as given in Table 12.
4. Add 26.8 μl of neat spermidine solution (9.296 g/l) to the base media.
5. Add amino acids in the same way of preparation of the CF media, see Table 3 for concentrations.
6. Add 1.5 g albumin, 1 g NaCl, 1.28 g of N-acetyl glucosamine, one at a time.
7. Sterilise the media in the same way to CF sinuses media (see protocol for CF sinuses)
8. Under sterile conditions, add 480 ml of autoclaved eDNA and 120 ml of autoclaved mucin to the mixture.
9. Adjust pH to 6.8-6.9 with 5 M NaOH and bring the volume to 1 litre with sterile water.
10. Filtered media should be stored at 4 °C for further use. Using fresh media is recommended, however it can be kept under these conditions for a maximum of three weeks. For freshness, the media can be aliquoted and stored at -80 °C (suitable for longer term use).

**Table 11.** Preparation of chemical stocks for healthy lungs media.

Chemical stock preparation for healthy lungs media			
Chemical	Stock concentration (mg/ml)	Amount of powder to weigh to form the stock solution (mg)	Volume of water to dissolve the powder in (ml)
Lysozyme	1	8	8
Lactoferrin	1	1	1
Spermine	1	1	1
Putrescine	200	200	1
CaCl _2_	250	250	1
MgCl _2_	100	100	1
FeCl _2_	1	5.95	5.95
CuCl _2_	1	1	1
ZnCl _2_	2.6	2.6	1
Sialic acid	1	1	1
Galactose	1	1	1
Succinate	80	320	4

**Table 12.** Volumes of each chemical to add into the base media for 1liter HLM.

Healthy lungs media			
Chemical	Stock concentration (mg/ml)	Concentration desired in the media	Amount to add from the stock
Lysozyme	1	8 μg/ml	8 ml
Lactoferrin	1	0.5 μg/ml	500 μl
Spermine	1	44.5 μg/l	44.5 μl
Putrescine	200	616 μg/l	3 μl
CaCl _2_	250	45 mg/l	180 μl
MgCl _2_	100	8 mg/l	80 μl
FeCl _2_	1	887 μg/L	887 μl
CuCl _2_	1	106 μg/L	106 μl
ZnCl _2_	2.6	390 μg/L	150 μl
Sialic acid	1	3.23 μg/L	3.23 μl
Galactose	1	6.27 μg/L	6.27 μl
Succinate	80	0.295 mg/ml	3.6 ml

**Incubation conditions for the media**

Incubation conditions differ between lung- and sinus media. The incubation conditions for each media are given in Table 13. Conditions of 5% CO _2_ can be achieved by incubating the media in GasPak Jars with candles, using Campygen packs or microaerobic incubators

**Table 13.** Incubation conditions for each developed media.

	Health sinuses	Healthy lungs	CF sinuses	CF lungs
Temperature	34 °C	37 °C	34 °C	37 °C
pH	6.8	6.8	6.8	6.8
CO _2_	Trace	~5%	Trace	~5%

**Notes on handling of media components and media preparation**

• Break bovine serum albumin into smaller pieces before adding to media, as it can take time to fully dissolve.
• Add eDNA and mucin to minimise clumping. Make sure that each addition is dissolved before adding more.
• Succinic acid may not fully dissolve in water immediately. Leave it at 37 °C for 30 minutes if needed.
• Polyamines should be handled in a fume hood due to toxicity at high concentrations. Read supplier instructions carefully.
• If a sterile pH probe cannot be used, we recommend aliquoting a small volume of media (2 ml) before each measurement, to maintain media sterility. Discard the aliquoted media after taking a measurement.
• As CF media is rich in chemicals, it is advised to use more than one filter unit, to prevent saturation.

## Results

### Media development

**Testing the effect of individual chemicals on bacterial growth**

For each chemical under consideration for inclusion in respiratory-mimicking media, the effect on *P. aeruginosa* growth was determined at three different concentrations. Experiments were performed over 20 hours of growth for PAO1 and LESB65, using M9 media as a base to support minimal bacterial growth (Underlying data Figures 1-4). PAO1 was chosen as a non-CF laboratory reference strain, to act as a comparator to the CF-adapted strain LESB65. Chosen concentrations of all chemicals were higher in CF conditions vs health conditions. Most chemicals were included in both health and CF conditions, with the exception of bile salts and glucose, which were only tested in CF conditions. Airway glucose is significantly elevated in CF related diabetes and bile in the lower airways is associated with CF, as a co-morbidity of gastroesophageal reflux disease. ^28^^,^^40^

Results from these experiments showed that, when used individually, most of the tested chemicals enhanced growth of both PAO1 and LESB65 (Underlying data Figures 1-4). The highest concentrations of bile salts, succinate and extracellular DNA (eDNA) used in CF conditions were toxic to bacteria (Underlying data Figures 1-4 (CF lung and CF sinus graphs)). The antimicrobial activity of lysozyme and lactoferrin was also observed to be both niche and concentration dependent, potentially due to decreased antimicrobial activity in lower temperatures, charge interference at high concentrations or induction of bacterial defence mechanism.

### Validation of pooled media use for phenotypic assays

**PAO1 and LESB65 can be maintained in respiratory-mimicking media for at least three days**

Next, the chosen concentration of each chemical was used to prepare pooled media. Growth of PAO1 and LESB65 was determined in the respiratory media, by CFU determination over 72 hours ( Figure 1). Data from these experiments show that viable PAO1 and LESB65 populations were maintained in all media for at least three days, with mid exponential growth of bacteria occurring at ~1×10 ^8^ CFU/ml ( Figure 1), similar to the findings of Palmer *et al.* for growth of *P. aeruginosa* in CF sputum. ^21^ PAO1 was able to grow to a higher density in CF sinus and lung media than in either of the equivalent conditions in health. In addition, PAO1 grew faster than LESB65 in all media and reached early stationary phase after 12-14 hours of growth, whilst LESB65 reached stationary phase only after 24 hours of growth in each media. Although LESB65 reached a similar density in all four media, CFU began to decrease in healthy sinus and lung media after 34 hours.

### Surface attached biofilm formation is niche and strain specific in sinus and lung media

Both attached and free-floating biofilm structures are thought to contribute to success of *P. aeruginosa* in the CF airways. Mature biofilms are commonly observed as free floating structures in mucus, whereas surface attached biofilms are suggested to dominate the early stages of infection, as physical contact of bacteria and epithelium is necessary as an initial survival strategy. ^41^^,^^42^ Crystal violet (CV) staining assays showed that there are niche, disease state and strain specific influences on biofilm formation ( Figure 2). PAO1 formed attached biofilms more readily than LESB65 in all airway niches and conditions. This may be due to the differences in growth rate of PAO1 and LESB65. In particular, PAO1 was able to form significantly more attached biofilm in sinus media compared to lung media. No statistically significant differences in biofilm formation were observed with LESB65 between healthy airway niches, but biofilm formed more readily in CF sinus media than in CF lung media. These results further highlight the importance of studying different airway niches and conditions in isolation. The media were compared with two widely-used laboratory media (LB and M9), and attached biofilm formation was shown to form most readily in LB.

### Developed media supports free-floating biofilm formation common in CF

Free-floating biofilm formation, associated with chronic infection, was assessed in respiratory-mimicking media, LB and M9, Figure 3. Free-floating biofilms formed more readily in LB and M9 than in any of the four respiratory media, for both isolates, suggesting that the respiratory media alter bacterial growth and biofilm formation, compared to standard laboratory media. Biofilms grew well in CF sinus and lung media, and in healthy lung media, but only minimal biofilm was observed in healthy sinus media. PAO1 outperformed LESB65 in the attached biofilm formation assays but not in the free-floating biofilm formation assays. As an airway adapted strain from chronic infection, LESB65 may be more adept at forming free-floating biofilms.

### CF media becomes viscous during infection, reflecting properties of CF sputum

Viscosity measurements were taken in the four developed media, pre and post biofilm formation ( Figure 4). Sterile HSM and HLM media were shown to have Newtonian liquid characteristics with very similar viscosity measurements to water (~1.0 mPa·s viscosity for water, 1.02 mPa·s for HSM and 1.03 mPa·s for HLM) ( Figure 4A). Viscosity changed only minimally in healthy media after bacterial growth (1.06 mPa·s for HSM and 1.05 mPa·s for HLM) ( Figure 4A). Both CF media were also determined to be Newtonian fluids pre-biofilm formation but had higher viscosity measurements compared to healthy media (1.37 mPa·s for CFSM and 1.27 mPa·s for CFLM) ( Figure 4A). When the biofilm was established in CF media, both media became more viscous, as compared to both their sterile controls and compared to post-biofilm formation healthy media. Infected CF media showed clear non-Newtonian fluid characteristics, with shear thinning properties (viscosity decreases as the shear rate increases) ( Figure 4B). Viscosity of CFSM media was approximately 16 mPa·s during infection at low shear rate ranges and decreased down to 1.57 mPa·s at the highest shear rate (1000 1/s). CFLM media was the most viscous media during infection, with approximately 2400 mPa·s viscosity at low shear rates, decreasing down to 1.8 mPa·s at 1000 1/s shear rate. These results are in agreement with previous literature, where the viscosity of CF sputum was shown to be higher than healthy controls. ^43^

### Airway mimicking media is stable for at least 30 days at 4°C.

**Figure 5.**
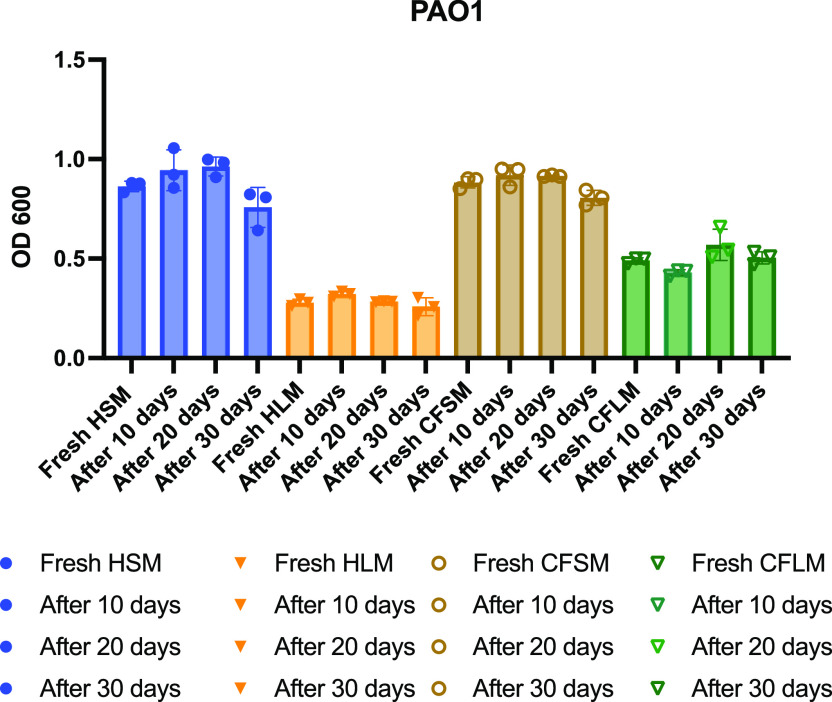
Stability of developed media over 30 days. Media stability was measured by testing biofilm formation of PAO1 in each developed media using CV stain assays. Each timepoint is one biological replicate representing the average of three technical replicates. No statistical significance was found between any of the fresh media vs the later treatments (One-way ANOVA *p<0.05, **p<0.01, ***p<0.001, ****p<0.0001).

Biofilm formation of PAO1 was measured over a month in the developed media stored at 4°C to test the stability of the airway mimicking media. Over a 30-day period, there was no significant change in the amount of biofilm formed by PAO1 in any of the four airway mimicking media ( Figure 5).

### Expression of infection-relevant genes in respiratory-mimicking media

**Figure 6.**
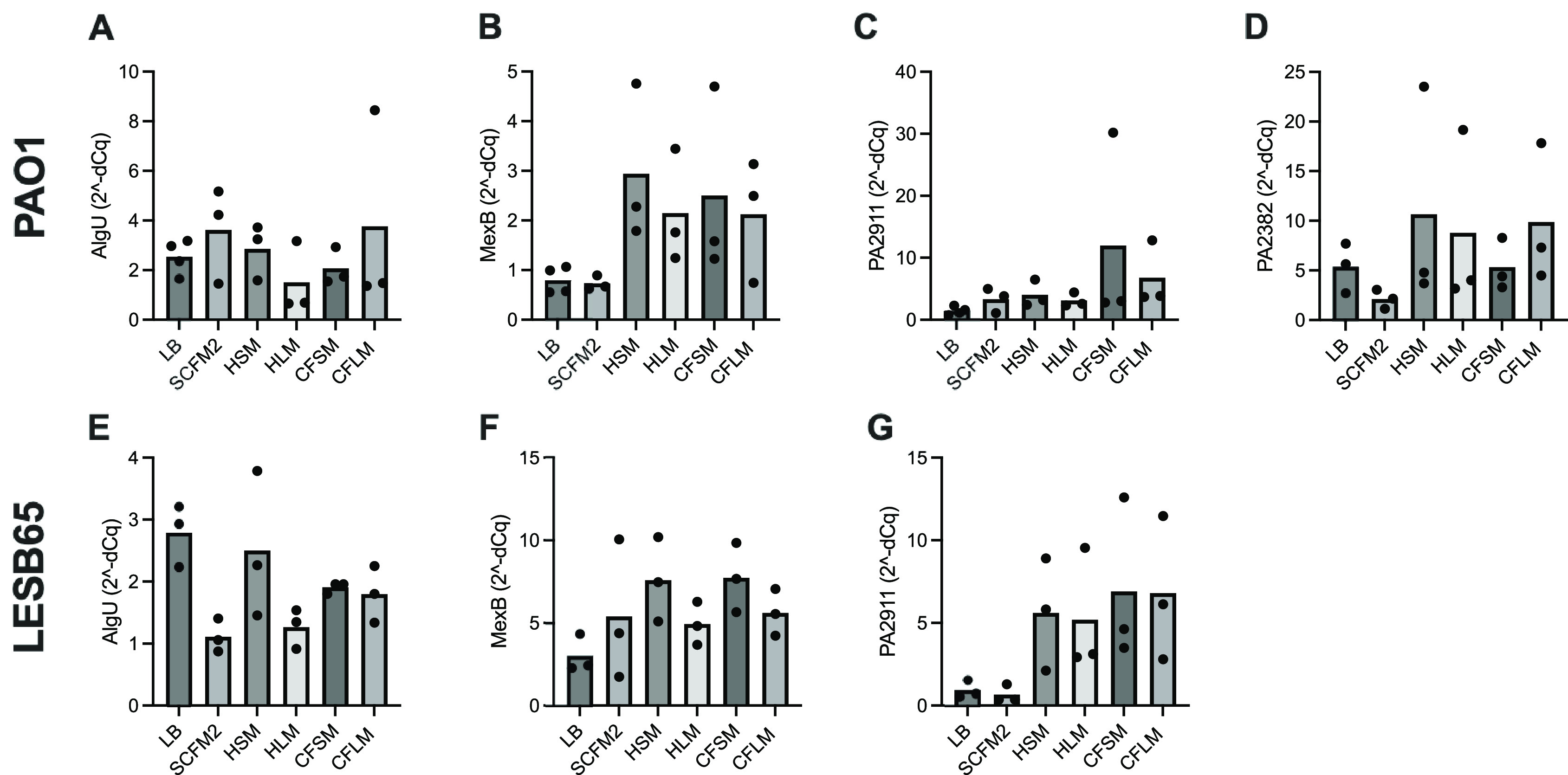
Expression of infection-relevant genes in respiratory media LB and SCFM2. Each point represents one biological replicate. Each biological replicate includes the mean of two technical replicates. RNA from bacteria grown in each media was extracted and used to synthesise cDNA. qPCR was performed using the cDNA by primers specific to A and E: algU, B and F: mexB, C and G: PA2911 and D: PA2382 (for which no expression was observed in LESB65 under any conditions). cDNA from LB was used as a negative (no treatment control). Statistical analysis: one-way ANOVA.

Four *P. aeruginosa* genes were selected, to determine whether growth in respiratory-mimicking media induced CF infection-relevant gene expression. The major virulence regulator, extracytoplasmic sigma factor *algU*, and *mexB* (part of a tripartite multidrug efflux system) genes were selected as they are virulence genes of *P. aeruginosa* known to contribute to chronic infection. ^44^^,^^45^ PA2911 and PA2382 genes were also selected, as expression of these genes in clinical isolates in CF sputum is observed but expression has not previously been captured in *in vitro* CF models. ^46^ *algU* is responsible for alginate overproduction, causing mucoidy and contributing to chronic infection in PwCF. ^44^ MexB is responsible for exporting biological metabolites and antimicrobial compounds, playing a crucial role in antimicrobial resistance in the airways. ^45^ PA2911 is predicted to be a Ton-B dependent receptor located in the outer membrane and responsible for sideophore transport. ^47^ PA2382 ( *lldA*) is a L-lactate dehydrogenase, important for pyruvate metabolism. ^48^ Results from qPCR studies showed that expression of all four genes were increased in all the airway-mimicking media when compared to expression in LB. Expression of the four genes was also determined in *P. aeruginosa* (PAO1 and LESB65) grown in SCFM2. Expression of *algU* and *mexB* was comparable in airway media and SCFM2, but expression of PA2911 and PA2382 was higher in respiratory-mimicking media than in SCFM2, although did not reach statistical significance, Figure 6.

### Long term culture of PAO1 in respiratory-mimicking media

**PAO1 growing in respiratory-mimicking media show changes in colony morphology**

**Figure 7.**
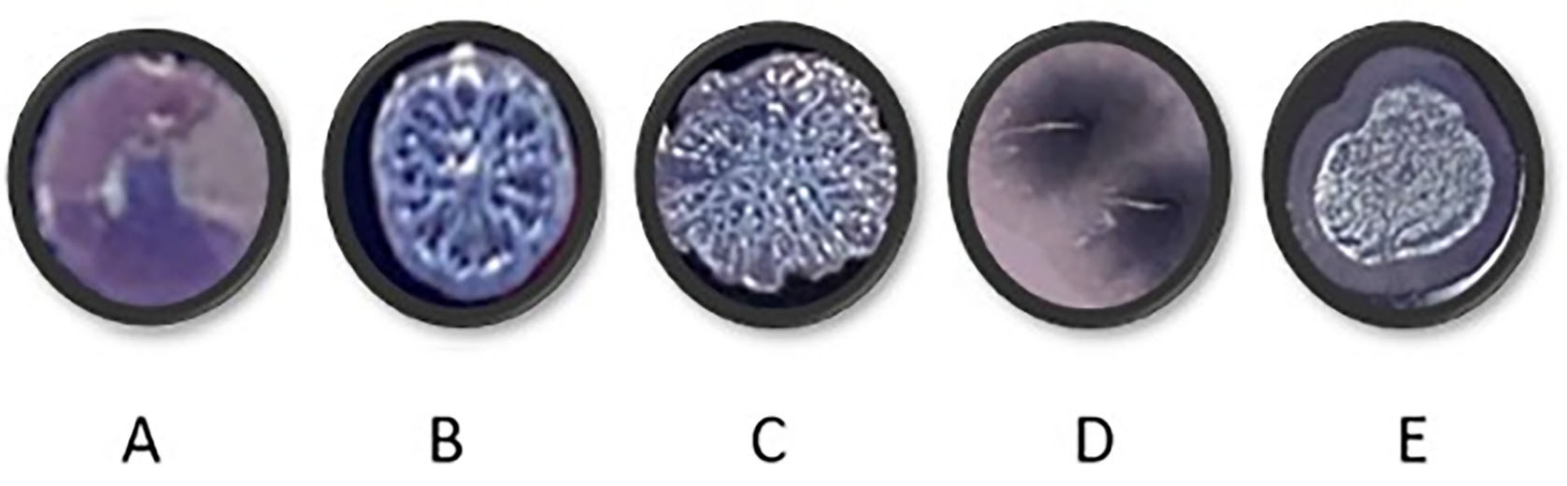
Representative colony morphologies observed during PAO1 culture over forty days ( Table 14). Cultures were streaked onto TCCA plates that contained Congo red and Coomassie blue to check for changes in colony morphologies. The plates were incubated at 37°C overnight and at room temperature for 48 hours before analysis. (B and C: wrinkly morphotypes, A: starting culture, D and E: colonies only observed in CF media.

PAO1 was grown for a period of 40 days in each of the four media. Every ten days, PAO1 populations cultured under different media conditions were streaked onto TCCA plates to observe colony morphotypes ( Figure 7). Only shiny, circular, concave colonies were observed in the starting populations. Wrinkly colonies began to appear in all media conditions over time. These wrinkly structures have been suggested to be the result of redox-driven adaptation that maximizes oxygen accessibility in biofilms, to increase their surface area in oxidant limitation. ^17^ Colony morphotypes in Figure 7D and E were visible only after transfer 10 and were unique to CF media ( Table 14).

**Table 14.** showing when each morphotype started to appear during long term culturing in each media per population.

Media	Day 0	Day 10	Day 20	Day 30	Day 40
HSM	A	A, B, C	A, B, C	A, B, C	A, B, C
HLM	A	A, B, C	A, B, C	A, B, C	A, B, C
CFSM	A	A, B, C, E	B, C, E, D	B, C, D	B, C, D
CFLM	A	A, B, C	A, B, C, E	A, B, C, E	A, B, C, E

## Discussion

Here we describe a simple suite of liquid culture models, designed to reproduce the conditions of upper and lower airway niches in CF and health. Our media have been designed to mimic the key properties of CF and healthy airways and have been validated for studying growth, as well as attached and free-floating biofilm formation of laboratory and CF-associated strains of *P. aeruginosa.* We previously showed that bacteria evolved within upper airways can acquire adaptive mutations conferring resistance to host-derived antimicrobials found in abundance in the lower airways. ^13^ Therefore, in this work, by inclusion of different host-derived antimicrobials in the media, we aimed to capture an important source of selective pressures acting on bacteria during airway infection. We show that bacteria can be maintained and cultured in these media long term, making them suitable for studying bacterial adaptation to airway environments using experimental evolution approaches in the laboratory. We envisage these media can help reduce the need for use of animals for the purposes of experimental evolution, investigation of host-pathogen interactions and for virulence screening.

To date, different liquid culture media have been developed with the aim of capturing the conditions of CF sputum. However, these media reflect the properties of lower airway regions. The growing evidence for the importance of the upper airways in shaping bacterial within-host adaptation makes liquid culture media reflecting sinus conditions a useful addition to the CF microbiologist’s toolkit. Having bespoke media for individual airway compartments allows airway niches to be studied in isolation, something impossible to do *in vivo.* To our knowledge, there are also no liquid culture media available that have been developed to mimic the conditions of healthy airways. In addition to the CF media, healthy sinus (HSM) and healthy lung (HLM) media will allow the research community to study other aspects of respiratory microbiology, such as bacterial sinusitis, or pneumonia.

In respiratory microbiology a common experimental approach is to screen panels of bacterial isolates in mouse models to determine virulence. ^49^^–^^51^ In one example study, researchers competed different pneumococcal serotypes in a mouse model of pneumococcal nasopharyngeal carriage. ^52^ The study used >200 mice, but many experiments could have been performed in sinus mimicking media, had it been available, with only the neutrophil-depletion studies necessitating animal usage. Thus, sinus mimicking media could have enabled 60-75% reduction in usage in the study. Carriage protocols are mild-severity, but similar studies to compare virulence in moderate or severe pneumonia models ^49^ could utilise lung mimicking media.

The culture media could be used to examine how bacterial communities develop in the face of host pressures, enabling study of biofilm formation in relevant environments. They offer a platform for assessment of bacterial gene expression in response to stress and to assess efficacy of drugs and therapeutics in a system more relevant than pathogen growth in broth, but more cost effective, less labour-intensive and ethical than animal models. The media can also be used as a pre-screening tool, to identify the most phenotypically interesting isolates to take forward into studies in more complex *in vitro* or *in vivo* systems, potentially leading to a significant reduction in the number of animals used to study bacterial pathogenicity in the host environment.

The developed media were designed to be cost effective for the purposes of long-term experiments ( Table 15) **.** The media should also prove readily accessible, as they can be prepared with standard laboratory equipment and without the need for any specialist training. The media can be easily modified to answer different research questions, for example by addition or removal of a metabolite or antimicrobial of interest. The media were also found to remain stable for 4 weeks at 4°C, although we recommend storing in -80°C for continued long term use.

**Table 15.** Costing of each media (per litre).

Media	Price per L
Healthy sinuses	£57.5
Healthy lungs	£61.5
CF sinuses	£214
CF lungs	£216

At concentrations associated with conditions of the healthy sinuses (Underlying data Figures 1 and 2), PAO1 showed increased growth in the presence of only two of the chemicals (eDNA and FeCl2), relative to the M9-only control. By contrast, at concentrations associated with CF sinuses (Underlying data Figures 1 and 2), significant increases in growth were observed for six of the chemicals (lactoferrin, mucin, eDNA, albumin, amino acids and FeCl2), relative to the M9-only control. Chemicals that induced higher growth rates at one or more of the concentrations tested for healthy and CF sinuses were albumin, eDNA, amino acids, iron, magnesium, copper, zinc and N-acetyl glucosamine (GlcNac). Many of these factors are well known to promote bacterial survival and may act as nutrient sources in these niches. ^21^^,^^53^^–^^55^ Iron and zinc are essential metals for bacteria. ^56^ Bacteria must actively acquire them through high affinity transport mechanisms. ^56^ In addition to these potential nutrient sources, the host derived antimicrobials lysozyme and lactoferrin were able to partially inhibit the growth of PAO1 at concentrations associated with health, however they did not cause significant growth inhibition at the higher concentrations of CF sinuses.

Similar to PAO1, increased growth of LESB65 was observed at CF-relevant concentrations for the majority of the chemicals. Growth was higher than the M9 control for at least one tested concentration of albumin, amino acids, mucin, spermine, zinc, lysozyme and lactoferrin. Relative to PAO1, the airway adapted isolate LESB65 appeared better able to grow in the face of high concentrations of host-derived antimicrobials and airway-abundant chemicals such as polyamines.

One of the key differences between CFSM and CFLM conditions was that the host-derived antimicrobial lactoferrin decreased the growth of PAO1 in CFLM while it increased the growth at all tested concentration in CFSM, where it may be acting as a nutrient source, as it is heavily glycosylated. ^57^ The enzymatic activity of lactoferrin will not function optimally at 34°C in sinuses media (SLM), nullifying its growth inhibiting activity.

Lysozyme had no significant positive or negative effect on growth of *P. aeruginosa* in CFLM but, in CFSM, all concentrations stimulated higher rates of growth. This may be a result of lysozyme-dependent changes in bacterial phenotype. Previously, it has also been suggested that the effectiveness of lysozyme is reduced in chronically infected airways due to electrostatic sequestration of the enzyme by anionic biopolymers. ^58^^,^^59^ Over production of inhibitor of vertebrate lysozyme (ivy) protein may also explain success in lysozyme-rich environments, as *ivy* knockout mutants show growth defects in the presence of lysozyme. ^60^ eDNA elevated bacterial growth in CFLM while it showed a growth inhibiting effect in HLM, where the concentrations tested were lower, further highlighting the positive effect of the chemically rich CF airways on bacterial survival.

Growth curves of PAO1 and LESB65 in airway media suggest a doubling time for PAO1 in CF media of 74 minutes (in both CFSM and CFLM), similar to the findings of *Yang et al.* for PAO1 in CF sputum (66 minutes). ^18^ The doubling time of PAO1 was ~85 minutes in healthy sinus and lung media. Doubling time of LESB65 was ~105 min in CFSM and ~92 minutes in CFL and ~86 minutes in both healthy media. This finding can be supported by the findings of *Yang et al.* who found two to threefold slower generation times in isolates from PwCF patients, compared to a laboratory strain. This observed reduced growth was also seen in other CF isolates, compared to non-CF isolates. ^18^ One possible explanation for this could be that the slow-growing phenotype develops as result of the stressful conditions associated with continuous exposure to host-derived antimicrobials and other molecules of the immune system. PwCF also undergo intensive antibiotic treatment, which acts as a further stress signal. Chromosomal mutations associated with antibiotic tolerance often also have growth rate-reducing effects. ^61^

Results from phenotypic assays show that airway media induce responses *in P. aeruginosa* that differ considerably from common laboratory and currently available liquid culture media (such as SCFM2), as evidenced by differences in attached and free-floating biofilm formation and expression of infection-relevant genes, including those that are highly expressed in CF sputum but poorly expressed in available liquid culture models. SCFM was developed to mimic the nutritional components of CF sputum and contains amino acids, ions, glucose and lactate ^21^ whereas HSM, HLM, CFSM and CFLM have been developed with the aim of not only capturing nutritional components but also the host derived antimicrobial content of different airway niches as well as stimulators of bacterial signalling systems, such as polyamines and bile salts.

An obvious limitation of these *in vitro* mimics is that they fail to capture the host cellular characteristics of different airway niches. The presence of immune cells significantly affects the pathophysiology of CF as a result of both the inflammatory processes and the direct effects of immune cells on bacteria. ^62^ Experiments combining the developed media with cell lines or primary cells (e.g., macrophages, epithelial cells) could provide additional information about host-pathogen interactions within the airways.

## Conclusion

In summary, we present here a collection of culture media replicating different airway niches in health and CF. Importantly, our study showed that these media can be used to study different phenotypic characteristics of bacteria or study of host-pathogen interactions. We show that the media can be used to study *P. aeruginosa*, a well-known CF pathogen, in health and disease by recapitulating different areas of the respiratory tract. With these compartmentalised airway-mimicking media, we aim to answer questions relating to how each airway niche affects bacterial behaviour and adaptation in CF and non-CF conditions, without the need for invasive whole animal studies for preliminary studies. We hope these media will become a resource for clinical microbiologists, respiratory clinicians, evolutionary biologists, pharmacologists and immunologists, as a tool to understand bacterial physiology, evolution, virulence and resistance to novel therapeutics, and as a means of reducing or replacing animal use.

**Table 16.** Materials and Equipment used in the study and supplier information.

Reagent	Supplier
Lactoferrin Human	Sigma-Aldrich (L4040)
Lysozyme Human	Sigma-Aldrich (L1667)
N-acetyl-glucosamine	Sigma-Aldrich (A8625)
Deoxyribonucleic acid from fish sperm	Sigma-Aldrich (74782)
Mucin from porcine stomach (Type II)	Sigma-Aldrich (M2378)
Bovine Serum Albumin	Sigma-Aldrich (A2153)
N-acetyl-neuraminic acid	Sigma-Aldrich (A0812)
Spermine	Sigma-Aldrich (S3256)
Spermidine	Sigma-Aldrich (S2626)
Putrescine	Sigma-Aldrich (51799)
CaCl _2_	Sigma-Aldrich (C5670)
MgCl _2_	Sigma-Aldrich (M8266)
CuCl _2_	Sigma-Aldrich (203149)
FeCl _2_	Sigma-Aldrich (372870)
ZnCl _2_	Sigma-Aldrich (229997)
Bile Salts	Sigma-Aldrich (B3426)
Succinate	Sigma-Aldrich (S9512)
Glucose	Sigma-Aldrich (G7021)
NaCl	Sigma-Aldrich (S9625)
Galactose	Sigma-Aldrich (G0750)
MgSO _4_	Sigma-Aldrich (M7506)
NaOH	Sigma-Aldrich (S5881)
L-Methionine	Sigma-Aldrich (M9625)
L-Phenylalanine	Across Organics (130310250)
L-Proline	Sigma-Aldrich (P0380)
L-Serine	Across Organics (132660250)
L-Threonine	Across Organics (138930250)
L(-)-Tryptophan	Across Organics (140590250)
L-Valine	Sigma-Aldrich (V0500)
L-Ornithine	Sigma-Aldrich (O2375)
L-Tyrosine	Across Organics (140641000)
L(+)-Asparagine monohydrate	Across Organics (175271000)
L-Alanine	Across Organics (102830250)
L-Arginine	Sigma-Aldrich (A5006)
L(+)-Aspartic acid	Across Organics (105041000)
L-Cysteine	Sigma-Aldrich (168149)
L-Glutamine	Sigma-Aldrich (G3126)
L-Glycine	Across Organics (220911000)
L-Histidine	Sigma-Aldrich (H8000)
L-Isoleucine	Sigma-Aldrich (I2752)
L-Leucine	Sigma-Aldrich (L8000)
L -Lysine	Sigma-Aldrich (L5501)
ME 2 diaphragm vacuum pump	Vacuubrand (696126)
Steritop filters (Pore size: 0.22 μm, Neck size: 45 mm)	EMD Millipore (SCGPT10RE)
U-bottomed 96-well microtiter plate	Greiner 650161
pH meter	HANNA instruments (HI-2550-02)
Petri dishes	Greiner (632180)
Parafilm	Starlab (I3080-1075)
LB broth	Neogen (NCM0088A)
LB agar	Neogen (NCM0142A)
Phosphate buffer saline	Sigma-Aldrich (P4417)
Muller Hinton Agar	Sigma-Aldrich (70191)
Tryptone Soy agar	Sigma-Aldrich (22091)
Congo red	Sigma-Aldrich (C6277)
Brilliant Blue	Sigma-Aldrich (27815)
Tryptone	Sigma-Aldrich (T7293)
BD Bacto™ dehydrate agar	Fisher Scientific (10455513)
Glass universal tubes	Fisher Scientific (14863562)
30ml universal tubes	Starlab (E1412-3010)
15ml Falcon tubes	Greiner (T1943)
50ml Falcon tubes	Greiner (T2318)
Sputasol	Thermo-Fisher (SR0233A)
Aspergillus cellulase	Sigma-Aldrich (22178)
Resazurin	Sigma-Aldrich (199303)
Crystal Violet	Sigma- Aldrich
Ceftazidime disks	Mast (CAZ30C)
Ciprofloxacin disks	Mast (CIP5C)
Doripenem disks	Mast (DOR10C)
Lexofloxacin disks	Mast (LEV5C)
Meropenem disks	Mast (MEM10C)
Cryovial beads	Pro-lab (16368776)
Direct-zol RNA Microprep kits	ZYMO Research (R2061)
TRI-reagent	ZYMO-Research (R2050)
Ultrapure nuclease free distilled water	Invitrogen (11538646)
iScript cDNA synthesis kit	BIO-RAD (1708891)
Fluostar omega microplate reader	BMG-Labtech (SPECTROstar Omega)
96 well programmable thermocycler	Applied Biosystem (4375786)
GoTaq qPCR Master Mix	Promega (A6001)
Primers	Eurofins
CFX Connect Real Time PCR System.	BIO-RAD (1855201)
Microamp Optical 96 well Reaction Plate	Applied Biosystem (N8010560)
Pseudomonas Selective Agar	Sigma-Aldrich (22470)
Citrate.H _2_O	VWR (BDH0288)
Ethanol	Sigma-Aldrich (51976)
Glycerol	Sigma-Aldrich (G5516)
KH _2_PO _4_	Sigma-Alrdrich (P0662)
Na _2_HPO _4_	Sigma-Aldrich (S9763)
NH _4_Cl	Sigma-Aldrich (213330)

## Data availability

### Underlying data

Figshare: Underlying data for “Development of liquid culture media mimicking the conditions of sinuses and lungs in cystic fibrosis and health”, https://doi.org/10.6084/m9.figshare.20463159.v6. ^81^

This project contains the following underlying data:

- Biofilm assay OD values and fluorescent intensity.xlsx
- F1000_qPCR_RawData.xlsx
- Growth CFUs.xlsx
- viscosity supplementary.xlsx
- Colony E fig 7.png
- colony D fig 7.png
- colony C fig 7.png

Data are available under the terms of the Creative Commons Attribution 4.0 International license (CC-BY 4.0).
