# Supplementary material for: Development of liquid culture media mimicking the conditions of sinuses and lungs in cystic fibrosis and health
Source: F1000Res. 2022 Nov 30;11:1007. Originally published 2022 Sep 7. [Version 2] doi: 10.12688/f1000research.125074.2 (PMC9718992; doi:10.12688/f1000research.125074.2)
Supplement: Peer review history

# Peer review: Reviewer response for version 2

**DOI:** 10.5256/f1000research.141209.r156986

**Published:** 2022-12-02

**Authors:** Ville-Petri Friman

**Affiliations:** 1 Department of Biology, University of York, York, UK

Some additional statistical analyses have now been included in the revised version. I have no further comments.

Is the rationale for developing the new method (or application) clearly explained?

Yes

Are the 3Rs implications of the work described accurately?

Yes

Is the description of the method technically sound?

Yes

If applicable, is the statistical analysis and its interpretation appropriate?

Partly

Are the conclusions about the method and its performance adequately supported by the findings presented in the article?

Yes

If any results are presented, are all the source data underlying the results available to ensure full reproducibility?

Yes

Are a suitable application and appropriate end-users identified?

Yes

Are sufficient details provided to allow replication of the method development and its use by others?

Yes

Reviewer Expertise:

Microbiology

I confirm that I have read this submission and believe that I have an appropriate level of expertise to confirm that it is of an acceptable scientific standard.

# Peer review: Reviewer response for version 1

**DOI:** 10.5256/f1000research.137340.r152082

**Published:** 2022-10-25

**Authors:** Sheyda Azimi, Jelly Vanderwoude

**Affiliations:** 1 School of Biological Sciences, Center for Microbial Dynamics and Infection, Georgia State University, Atlanta, GA, USA; 2 Georgia Institute of Technology, Atlanta, GA, USA

In this article, the authors sought to develop four new culturing media—Healthy Sinus Media (HSM), Healthy Lung Media (HLM), Cystic Fibrosis Sinus Media (CFSM), and

 Cystic Fibrosis Lung Media (CFLM)—to recapitulate the environment of human lungs and sinuses, both in healthy individuals and those with cystic fibrosis (CF). The authors justify the need for the development of novel media by emphasizing that Artificial Sputum Media (ASM) and Synthetic Cystic Fibrosis Media (SCFM and SCFM2) capture many key nutritional aspects of the CF lung, they lack critical host-derived antimicrobials, polyamines, glucose, and bile salts. All of which impact *Pseudomonas aeruginosa* growth. Furthermore, there are yet no media that have been developed to capture the environments of healthy human lungs or sinuses, nor that of CF-impacted sinuses.

 This article is scientifically sound in developing and thoroughly testing four novel media to model human airways. The authors employ many different experimental tests to assay the viability of these novel media and assess their relevance to *P. aeruginosa *infection, including but not limited to: culturing strains in each chemical component independently before pooling, measuring growth and biofilm phenotypes, assessing long-term media stability, measuring gene expression, etc.

 The authors use appropriate statistical tests as necessary and conduct all experiments with technical and biological replicates. In addition, the authors describe detailed methods for preparing each medium, including supplier information for all reagents, stock concentrations, volumes, final concentrations, sterilization procedures, and special notes on media preparation so that others may easily reproduce these procedures.

 The authors provide a rationale for each component added with references to literature. The authors conduct all experiments in appropriate conditions to mimic *in vivo*, for example, altering growth temperatures between sinus and lung media. The article also provides sufficient rationale for the necessity of these media in the scientific community. The creation of these novel media will significantly add to the scientific community’s ability to better model CF infection dynamics *in vitro* preclinical models. Including healthy lung and sinus media will no doubt serve as a useful control for these experiments than LB or M9. Having accurate models for upper airway infection is also key in understanding the intermittent colonization and persistence of *P. aeruginosa* during initial colonization and antibiotic treatment.

 The various components tested by the authors for the development of these novel media were: Lysozyme, Lactoferrin, Spermidine, Spermine, Putrescine, Mucin, eDNA,

 Albumin, CaCl2, MgCl2, FeCl2, CuCl2, ZnCl2, NaCl, Sialic acid, Bile, N-acetyl glucosamine, Glucose, Succinate, Galactose, L-Methionine, L-Phenylalanine, LProline,

 L-Serine, L-Threonine, L-Tryptophan, L-Valine, L-Ornithine, L-Tyrosine, L(+)-Asparagine monohydrate, L-Alanine, L-Arginine, L(+)-Aspartic acid, L-Cysteine, LGlutamine,

 L-Glycine, L-Histidine, L-Isoleucine, L-Leucine, and L-Lysine. The authors chose the PAO1 and LESB65 as representative laboratory and CF-adapted strains of *P. aeruginosa* in their tested conditions. The authors first grew these strains in M9 media supplemented with each chemical component independently at three different concentrations. These concentrations were selected to most closely reflect the physiological concentration of each compound within each niche, based upon cited references in the literature or experimental testing of metabolites. Upon confirmation that these strains could grow sufficiently at the selected concentration of each metabolite, the authors then pooled the components at the appropriate ratios to yield four novel respiratory media: Healthy Sinus Media (HSM), Healthy Lung Media (HLM), Cystic Fibrosis Sinus Media (CFSM), and Cystic Fibrosis Lung Media (CFLM).

 The authors tested the viability and relevance of these media as preclinical models for human respiratory infections by assessing the:

 i) growth and biofilm formation of strains cultured continuously for 72 hours;

 ii) viscosity and shear rate in the media pre- and post-biofilm formation;

 iii) the stability of media after storage at 4° C for 40 days;

 iv) transcriptional changes of infection-relevant genes; and

 v) changes in colony morphology of strains evolved for 40 days;

 of PAO1 and LESB65 in each medium.

 The growth rate experiments show that PAO1 and LESB65 could be maintained for at least 72 hours, with similar dynamics to those previously published for *P. aeruginosa* in SCFM (authors may include the publications that used these media to study various aspects of *P. aeruginosa* infection). The crystal violet assays, which included growth in M9 and Luria-Bertani (LB) as additional comparators against the newly described media, show niche, disease state, and strain-specific differences in surface-attached and free-floating biofilm formation, highlighting the importance of using clinically-relevant models for estimating biofilm formation. Although the aggregate and aggregate formation are not tested here, authors can discuss those future assessments for these media.

 The authors also show that the viscosity and shear rate measurements of their proposed media agree with those published previously for CF and healthy human sputum. The authors also show that each media could support the same level of biofilm formation in PAO1 after 0, 10, 20, and 30 days of storage at 4° C, implying that the media remains stable for this duration. qPCR analysis shows that expression of the four tested virulence genes—algU, mexB, PA2911, and PA2382—significantly increased in all four novel media compared to their transcription levels in LB and additionally were comparable to transcription levels of the same strains grown in SCFM2. Long-term culturing of PAO1 in these media shows the evolution of a wrinkly colony morphotype, a potential redox-driven adaptation to maximize oxygen availability in biofilms, and morphological adaptations specific to CF media.

**Some minor comments:**

 The authors determined these novel media's viability over 30 days by measuring PAO1 biofilm formation at 0, 10, 20, and 30 days. While it is nice to show that the media sustain similar levels of biofilm growth regardless of whether the media is freshly prepared, this might not be the best indicator of media stability over long periods. Instead, it would have been nice to see the authors conduct LCMS analyses to determine the stability of each of the chemical components at 0 and 30 days and detect if any possible degradation had occurred. Also, it is important to determine whether the growth of *P. aeruginosa* in the stored media displays any altered metabolism, physiology, or spatial organization.

 Additionally, instead of hand-picking a short list of four genes, it would have greatly strengthened the supporting case for these novel media by RNA-seq analysis. For example, comparing the entire transcriptional profile of *P. aeruginosa* grown in new media (fresh and stored at 4° C) to the transcriptional profile of cells grown in SCFM2 and *in vivo* samples would confirm the similarity of these preclinical models to the infection and healthy environments.

 I also understand that these experiments can be addressed in the future and are out of the scope of this study, which aimed to introduce these novel media for upper and lower respiratory tracts; however, I recommend that authors address this in the discussion section. I recommend authors include these studies and discuss the key benefits of using the novel HSM, CFSM, HLM, and CFLM media over the other versions available.

 In the section titled “Airway mimicking media is stable for at least 30 days at 4° C,” the wording of the first sentence leads to some confusion. At first glance, it appears to imply that PAO1 was cultured for 30 days, although the authors meant to say that PAO1 was cultured in media that had been stored for 30 days. The authors may want to re-write this sentence to avoid confusion.

 In Tables 3, 8, and 12, please use a consistent format for the concentration units μg/L or μg/l.

 In Figure 5, the legend below the chart is redundant, as it shows the same data as the labels on the X-axis. I would recommend removing this legend to simplify the readers' view.

 In Figure 6, standard deviation bars and significance labels would help the reader understand which relationships are significant without having to reference the text.

 There are studies on the evolution of *P. aeruginosa* in TSB, SCFM, and SCFM2 by Schick A, Kassen R (2018) ^1^, Azimi *et al., *2020 ^2^, and Flynn *et al.,* 2016 ^3^, showing the emergence of various morphotypes in the populations that authors may want to include.

 The emergence of the observed morphotypes and phenotypes in this study is similar to what is observed as a result of evolving *P. aeruginosa *in other preclinical infection models are not unique to novel developed media. I suggest that authors include previous studies, discussing how future studies of genetic and phenotypic adaptation to the developed media are required. Thus, future analysis can confirm the unique adaptive traits observed in this study, similar to upper and lower CF respiratory infections.

 In Table 14, I believe the title is somewhat of a misnomer. The table shows when each morphotype appears and whether it is maintained in the populations. I would consider re-titling this table to reflect the prevalence of each morphotype over the evolution period.

Is the rationale for developing the new method (or application) clearly explained?

Yes

Are the 3Rs implications of the work described accurately?

Yes

Is the description of the method technically sound?

Yes

If applicable, is the statistical analysis and its interpretation appropriate?

Yes

Are the conclusions about the method and its performance adequately supported by the findings presented in the article?

Partly

If any results are presented, are all the source data underlying the results available to ensure full reproducibility?

Yes

Are a suitable application and appropriate end-users identified?

Yes

Are sufficient details provided to allow replication of the method development and its use by others?

Yes

Reviewer Expertise:

Chronic infections, Biogeography of Infection, Pseudomonas aeruginosa population dynamics.

We confirm that we have read this submission and believe that we have an appropriate level of expertise to confirm that it is of an acceptable scientific standard.

## References

[1] : Rapid diversification ofPseudomonas aeruginosa in cystic fibrosis lung-like conditions. *Proceedings of the National Academy of Sciences* .2018;115(42) : 10.1073/pnas.1721270115 10714-10719 10.1073/pnas.1721270115 PMC619650730275334

[2] : Allelic polymorphism shapes community function in evolving Pseudomonas aeruginosa populations. *ISME J* .14(8) : 10.1038/s41396-020-0652-0 1929-1942 10.1038/s41396-020-0652-0 32341475PMC7368067

[3] : Evolution of Ecological Diversity in Biofilms of Pseudomonas aeruginosa by Altered Cyclic Diguanylate Signaling. *Journal of Bacteriology* .2016;198(19) : 10.1128/JB.00048-16 2608-2618 10.1128/JB.00048-16 PMC501905227021563

# Response

**Published:** 2022-11-18

**Authors:** DILEM RUHLUEL

**Affiliations:** University of Liverpool, UK

Thank you for the positive comments regarding the manuscript and for your helpful suggestions for improvements and discussion points. We have addressed each point raised, below and have submitted an updated manuscript version in which the changes have been made.

*The authors determined these novel media's viability over 30 days by measuring PAO1 biofilm formation at 0, 10, 20, and 30 days. While it is nice to show that the media sustain similar levels of biofilm growth regardless of whether the media is freshly prepared, this might not be the best indicator of media stability over long periods. Instead, it would have been nice to see the authors conduct LCMS analyses to determine the stability of each of the chemical components at 0 and 30 days and detect if any possible degradation had occurred. Also, it is important to determine whether the growth of P. aeruginosa in the stored media displays any altered metabolism, physiology, or spatial organization.*

 We agree both that LCMS would offer a more complete determination of chemical stability and that investigation of bacterial metabolism, physiology and spatial organisation would be desirable. We have added a sentence to the discussion, highlighting the need for further studies into media stability. These studies are underway in our lab.

*Additionally, instead of hand-picking a short list of four genes, it would have greatly strengthened the supporting case for these novel media by RNA-seq analysis. For example, comparing the entire transcriptional profile of P. aeruginosa grown in new media (fresh and stored at 4° C) to the transcriptional profile of cells grown in SCFM2 and in vivo samples would confirm the similarity of these preclinical models to the infection and healthy environments.*

*I also understand that these experiments can be addressed in the future and are out of the scope of this study, which aimed to introduce these novel media for upper and lower respiratory tracts; however, I recommend that authors address this in the discussion section. I recommend authors include these studies and discuss the key benefits of using the novel HSM, CFSM, HLM, and CFLM media over the other versions available.*

 We completely agree and these studies have already begun. The large RNAseq dataset will require careful analysis. We feel that the space and word count needed to describe those data fully would make the current manuscript overly lengthy. As the reviewers suggest, we have instead mentioned the need for these investigations in the discussion.

*In the section titled “Airway mimicking media is stable for at least 30 days at 4° C,” the wording of the first sentence leads to some confusion. At first glance, it appears to imply that PAO1 was cultured for 30 days, although the authors meant to say that PAO1 was cultured in media that had been stored for 30 days. The authors may want to re-write this sentence to avoid confusion.*

 Sorry for this confusion, we have now reworded the sentence.

*In Tables 3, 8, and 12, please use a consistent format for the concentration units μg/L or μg/l.*

 We have now used μg/l throughout.

*In Figure 5, the legend below the chart is redundant, as it shows the same data as the labels on the X-axis. I would recommend removing this legend to simplify the readers' view.*

 This figure has been reworked and the legend removed.

*In Figure 6, standard deviation bars and significance labels would help the reader understand which relationships are significant without having to reference the text.*

 Standard deviation bars and significance labels have now been added to these graphs and we have described ANOVA summary statistics in the main text.

*There are studies on the evolution of P. aeruginosa in TSB, SCFM, and SCFM2 by Schick A, Kassen R (2018) 1* *, Azimi et al., 2020 2* *, and Flynn et al., 2016 3* *, showing the emergence of various morphotypes in the populations that authors may want to include.*

 Thank you for highlighting this. Reference to these studies has been added to our discussion.

*The emergence of the observed morphotypes and phenotypes in this study is similar to what is observed as a result of evolving P. aeruginosa in other preclinical infection models are not unique to novel developed media. I suggest that authors include previous studies, discussing how future studies of genetic and phenotypic adaptation to the developed media are required. Thus, future analysis can confirm the unique adaptive traits observed in this study, similar to upper and lower CF respiratory infections.*

 Thank you for this suggestion. We have added the three references mentioned in the previous comment, alongside some discussion of the need to deconvolute those phenotypes that are adaptive for different respiratory environments from those that are merely adaptive to different modes of growth.

*In Table 14, I believe the title is somewhat of a misnomer. The table shows when each morphotype appears and whether it is maintained in the populations. I would consider re-titling this table to reflect the prevalence of each morphotype over the evolution period.*

 We have re-titled this table. Unfortunately, these data were captured as presence/absence for each morphotype, at each time point. We don’t, therefore, have information on relative or absolute morphotype abundance over time.

# Peer review: Reviewer response for version 1

**DOI:** 10.5256/f1000research.137340.r151644

**Published:** 2022-10-25

**Authors:** Ville-Petri Friman

**Affiliations:** 1 Department of Biology, University of York, York, UK

This is a very nice manuscript describing four new synthetic media mimicking growth conditions in the human lung. The main advancement is the development of both CF and healthy lung media and the addition of host-derived antimicrobials and stimulators (bile salts and polyamines) of bacterial signalling systems that are not included in commonly used SCFM2 medium. To some extent, this comparison falls a bit short as not all measurements are performed in SCFM2 and developed media. Moreover, the gene expression analysis relies on the expression of a few marker genes instead of whole genome transcriptomics, which could have revealed further interesting differences and allowed linking data with available transcriptomics data from CF lungs. Nevertheless, these are small omissions and could perhaps just be discussed briefly in the discussion.

 My main concern is the statistical analysis that could be improved a bit. I would suggest using full factorial analysis to clearly show if growth in the media is different for the two studied strains and if there are any interesting interactions between strain identity and specific media. These statistics should be properly reported in the text or supplementary tables including test statistics, error, and degrees of freedom.

 I would suggest:

- Use Repeated Measures Anova or GLMMs for analysing time-dependent data in Figure 1.
- Include strain identity in the model in Figures 2-3 to statistically compare if strains grew differently in these media.
- Figure 4: Which bacteria were grown in these media before the measurements? Or is this data averaged over both bacteria? Needs a little bit more information.
- Figure 5: Could also analyse the effect of time for each media and across all media to show that no change in biofilm formation was observed. I would reconsider naming this measure as ‘Stability of biofilm formation’ as this is what you measure instead of media stability, which could be understood as a measure of degradation of its components. Just to avoid confusion.
- Figure 6: I think the results here are much more nuanced than described in the results text. For example, the expression of algU is clearly different from other marker genes and there is no clear increase in its expression relative to growth in LB media (in contrast to what is said in the results text). Here, factorial Anova (or GLMM) would be powerful to show significant interactions between different media, studied strains, and developed media.
- Figure 7 and Table 14: Do you have quantitative data for colony morphotype frequencies during the experiment? Data is now quite descriptive.
- Out of interest, did you specifically test the effect of freezing (-80°C) for media composition?

Is the rationale for developing the new method (or application) clearly explained?

Yes

Are the 3Rs implications of the work described accurately?

Yes

Is the description of the method technically sound?

Yes

If applicable, is the statistical analysis and its interpretation appropriate?

Partly

Are the conclusions about the method and its performance adequately supported by the findings presented in the article?

Yes

If any results are presented, are all the source data underlying the results available to ensure full reproducibility?

Yes

Are a suitable application and appropriate end-users identified?

Yes

Are sufficient details provided to allow replication of the method development and its use by others?

Yes

Reviewer Expertise:

Microbiology

I confirm that I have read this submission and believe that I have an appropriate level of expertise to confirm that it is of an acceptable scientific standard, however I have significant reservations, as outlined above.

# Response

**Published:** 2022-11-18

**Authors:** DILEM RUHLUEL

**Affiliations:** University of Liverpool, UK

Thank you for the helpful comments and suggestions for alternative data analysis. We have addressed each point raised, below and have submitted a revised manuscript in which the indicated changes have been made.

*To some extent, [the phenotypic] comparison falls a bit short as not all measurements are performed in SCFM2 and developed media. Moreover, the gene expression analysis relies on the expression of a few marker genes instead of whole genome transcriptomics, which could have revealed further interesting differences and allowed linking data with available transcriptomics data from CF lungs. Nevertheless, these are small omissions and could perhaps just be discussed briefly in the discussion.*

 We agree that transcriptomic data would represent an important validation dataset for these media. We have now added some discussion of this point to the manuscript and hope to be able to report these data shortly. We agree, also, that it would be preferable to have SCFM2 data for all phenotypic comparisons. Where these are absent, we have now referenced studies conducted by others, in which SCFM2 was used.

*My main concern is the statistical analysis that could be improved a bit. I would suggest using full factorial analysis to clearly show if growth in the media is different for the two studied strains and if there are any interesting interactions between strain identity and specific media. These statistics should be properly reported in the text or supplementary tables including test statistics, error, and degrees of freedom.*

*I would suggest:*

*Use Repeated Measures Anova or GLMMs for analysing time-dependent data in Figure 1.*

 We were cautious of over-interpreting these data, particularly when comparing between strains, as PAO1 and LESB65 have considerable differences in growth profile, even in standard laboratory media. However, we agree with the reviewer that comparisons of relative growth between media would be informative. We have now included empirical area under the curve (AUC_e) values for both strains, in all four media. These were determined from the original growth data in Figure 1. Two-way ANOVA analysis (alpha 0.05) of the AUC_e values shows that both strain and media type are significant variables (p < 0.0001, with 3 df for media type, p = 0.0084, with 1 df for strain). Sidak’s multiple comparison testing demonstrates the success of PAO1 in the CF media, relative to in healthy sinus and lung media. P values and a description of the findings have been added to the manuscript text and the AUC_e values are presented in Figure 1C.

*Include strain identity in the model in Figures 2-3 to statistically compare if strains grew differently in these media.*

 We have done as suggested and provide updated figures, analysis and text in the revised manuscript. Strain was found to be a significant variable in both biofilm assays. We have reported summary statistics and pairwise comparisons in the text. Briefly, for crystal violet assays, PAO1 formed attached biofilms more readily than LESB65 in all airway niche media bar HLM (p < 0.0001 vs LESB65 in HSM, CFSM and CFLM, p > 0.9999 in HLM in two-way ANOVA with Sidak’s multiple comparisons test). In resazurin assays, PAO1 biofilm formation differed significantly from that of LESB65 in HLM (p = 0.0001) and HSM (p < 0.0001) but not in the other media tested.

*Figure 4: Which bacteria were grown in these media before the measurements? Or is this data averaged over both bacteria? Needs a little bit more information.*

 This was done with PAO1. We have added this info to the figure legend and the text in the results section.

*Figure 5: Could also analyse the effect of time for each media and across all media to show that no change in biofilm formation was observed.*

 We have re-analysed with an ordinary two-way ANOVA. Media is a significant factor (P<0.0001), whilst media storage time is not (P=0.4174). No individual pairwise comparisons between timepoints proved significant for any of the four media. We have reported the ANOVA summary statistics in the text, as suggested.

*I would reconsider naming this measure as ‘Stability of biofilm formation’ as this is what you measure instead of media stability, which could be understood as a measure of degradation of its components. Just to avoid confusion.*

 The title has been changed, as suggested.

*Figure 6: I think the results here are much more nuanced than described in the results text. For example, the expression of algU is clearly different from other marker genes and there is no clear increase in its expression relative to growth in LB media (in contrast to what is said in the results text). Here, factorial Anova (or GLMM) would be powerful to show significant interactions between different media, studied strains, and developed media.*

            

 Apologies, our interpretation of these data was overly simplistic. We have now performed two-way ANOVA for each gene, to assess the contribution of strain and media type to observed differences. We have reported these in the manuscript, along with updated graphs and data descriptions. Briefly, strain was found to be a significant variable for *mexB *and *PA2382 *expression (p < 0.0001 for both), whilst media type was a significant variable for *mexB *(p = 0.0308) and *PA2911* (p = 0.0462) expression. In Dunnett’s multiple comparison test, with LB set as the control group for each strain, significant differences in gene expression were identified for *mexB *with LESB65 grown in HSM (p = 0.0249) or CFSM (p = 0.0201) and for *PA2382 *with PAO1 grown in CFSM (p = 0.0243) or CFLM (p = 0.0273). We agree that *algU *is clearly different from the other genes and have acknowledged this in the manuscript text.

*Figure 7 and Table 14: Do you have quantitative data for colony morphotype frequencies during the experiment? Data is now quite descriptive.*

 Unfortunately not. We plated a proportion of the total growth at each transfer and then recorded the different colony morphotypes as present or absent, rather than a quantitative measure.

*Out of interest, did you specifically test the effect of freezing (-80°C) for media composition?*

 Only in the same way as reported for the 4°C storage, i.e. we confirmed that the media would still support biofilm growth. We’ve added to the discussion that LCMS could provide additional useful data on media stability under different conditions.
